# Structural basis of lipoprotein recognition by the bacterial Lol trafficking chaperone LolA

**DOI:** 10.1073/pnas.2208662119

**Published:** 2022-08-29

**Authors:** Elise Kaplan, Nicholas P. Greene, Abigail E. Jepson, Vassilis Koronakis

**Affiliations:** ^a^Department of Pathology, University of Cambridge, Cambridge CB2 1QP, United Kingdom

**Keywords:** Lol lipoprotein trafficking, protein–lipid interaction, X-ray crystallography, ABC transporter, antibacterial target

## Abstract

In gram-negative bacteria, lipoproteins are vital structural components of the outer membrane (OM) and crucial elements of machineries central to the physiology of the cell envelope. A dedicated apparatus, the Lol system, is required for the correct localization of OM lipoproteins and is essential for viability. The periplasmic chaperone LolA is central to this trafficking pathway, accepting triacylated lipoproteins from the inner membrane transporter LolCDE, before carrying them across the periplasm to the OM receptor LolB. Here, we report a crystal structure of liganded LolA, generated in vivo, revealing the molecular details of lipoprotein association. The structure highlights how LolA, initially primed to receive lipoprotein by interaction with LolC, further opens to accommodate the three ligand acyl chains in a precise conformation within its cavity. LolA forms extensive interactions with the acyl chains but not with any residue of the cargo, explaining the chaperone’s ability to transport structurally diverse lipoproteins. Structural characterization of a ligandedLolA variant incapable of lipoprotein release reveals aberrant association, demonstrating the importance of the LolCDE-coordinated, sequential opening of LolA for inserting lipoprotein in a manner productive for subsequent trafficking. Comparison with existing structures of LolA in complex with LolC or LolCDE reveals substantial overlap of the lipoprotein and LolC binding sites within the LolA cavity, demonstrating that insertion of lipoprotein acyl chains physically disengages the chaperone protein from the transporter by perturbing interaction with LolC. Taken together, our data provide a key step toward a complete understanding of a fundamentally important trafficking pathway.

## Significance

Lipoproteins in gram-negative bacteria underpin the formation and maintenance of the outer membrane that constitutes a vital protective barrier against antibiotics and other noxious molecules. An essential transport system comprising the LolABCDE proteins is required to traffic lipoproteins to the outer membrane. Following maturation on the inner membrane and extraction by the LolCDE transporter, lipoproteins are passed to the chaperone LolA that carries them across the periplasm prior to insertion into the outer membrane by the LolB receptor. Here, we report the molecular details of lipoprotein interaction with the chaperone LolA, a key intermediate located at the heart of the Lol pathway. The structure provides valuable insights into this important system and could be exploited to develop new antimicrobials.

The outer membrane (OM) of gram-negative bacteria surrounds the inner membrane and intervening periplasm, protecting the cell against osmotic shock and noxious compounds, including antibiotics (1). Lipoproteins, anchored by an N-terminal triacyl group, are essential components of this barrier underlying its structural integrity and forming vital components of machineries essential for lipopolysaccharide (LPS) insertion, outer membrane protein assembly, and maintenance of its asymmetry (2–4). They also underpin a myriad of other vital functions, including nutrient acquisition, stress sensing, and bacterial virulence (5, 6), rendering the systems involved in their synthesis and transport key targets for antimicrobial therapy (7, 8). Lipoproteins are initially produced in the cytosol and targeted by an N-terminal signal peptide to the inner membrane where they are transported by the Sec or Tat pathways (9, 10). The lipobox, a four-residue motif at the signal peptide C terminus, then directs the progressive modification of an invariant cysteine by three inner membrane enzymes. First, the thiol side chain is diacylated by Lgt before the signal peptide is cleaved by Lsp. Finally, Lnt acylates the N-terminal amine of the cysteine residue, resulting in a mature triacylated lipoprotein anchored in the outer leaflet of the inner membrane (10, 11).

With the exception of a subset of lipoproteins that carry a transport avoidance signature and remain in the inner membrane (12–14), the majority of lipoproteins are destined for the outer membrane. Their transport is directed by the Lol system that comprises the inner membrane transporter LolCDE, the periplasmic chaperone LolA, and the outer membrane receptor LolB, itself a lipoprotein (10). All these genes are essential in wild-type (WT) *Escherichia coli* (15–18), principally because of the deleterious effects of Lpp mislocalization in the inner membrane (19).

**Fig. 1.**
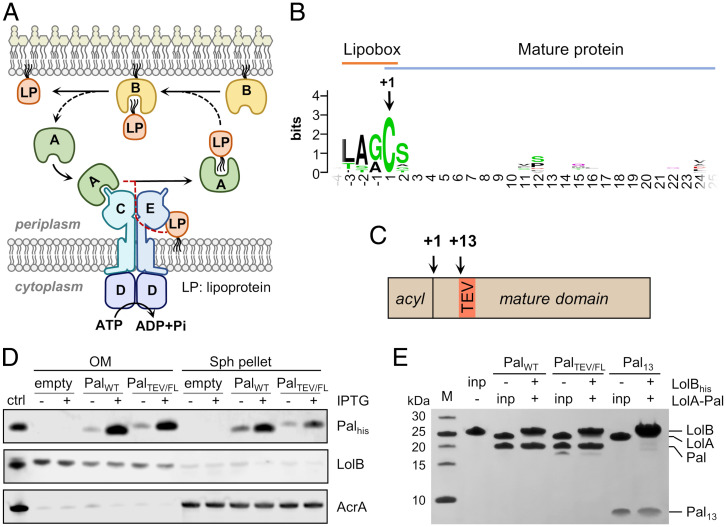
Isolation and trafficking of a modified Pal lipoprotein amenable to structural study. (*A*) Lipoprotein trafficking in *E. coli* mediated by the LolABCDE proteins. Lipoproteins are extracted from the inner membrane by LolCDE, a process driven by hydrolysis of adenosine triphosphate (ATP) to adenosine diphosphate (ADP) and inorganic phosphate (Pi). Following transfer to LolA, lipoproteins are transported across the periplasm prior to outer membrane insertion by LolB. (*B*) Conservation plot of the N-terminal region of selected *E. coli* lipoproteins (sequences and Uniprot entry codes are listed in *SI Appendix*, Fig. S1). The invariant, +1 triacylated cysteine is highlighted as are conserved residues forming the lipobox. (*C*) Schematic representation of Pal_TEV/FL_ lipoprotein indicating the relative positions of the TEV cleavage site and the +1 cysteine. (*D*) Immunoblot showing the cellular localization of His-tagged Pal_WT_ and Pal_TEV/FL_. *E. coli* C43 (DE3) cells bearing an empty vector, LolA–Pal_WT_ or LolA–Pal_TEV/FL_ plasmid were induced (+) or not (−) with isopropyl β-D-thiogalactoside (IPTG) and converted into spheroplasts. Outer membrane (OM) fraction was recovered from the supernatant, and spheroplast (Sph) pellets were subjected to immunoblotting with anti-His antibodies. Detection of triacylated LolB and AcrA was performed as outer and inner membrane integrity controls, respectively. Purified Pal, LolB and AcrA proteins served as controls (ctrl) for the antisera. (*E*) In vitro transfer of Pal_WT_, Pal_TEV/FL_, and Pal_13_ from LolA to mLolB. Input purified proteins were loaded as a benchmark (inp). LolA–Pal proteins were mixed with His-tagged mLolB and loaded on IMAC resin. After several washes, bound proteins were eluted and analyzed by SDS-PAGE. Molecular masses of protein standards (M) are indicated.

Lipoproteins are first extracted from the inner membrane by the type VII ABC transporter LolCDE, which comprises a dimer of the ATPase LolD and a heterodimer of LolC and LolE, the transmembrane subunits. LolC and LolE are homologous in structure, composed of four transmembrane helices and a helical stalk that elevates a globular periplasmic domain above the membrane, but functionally distinct (20, 21). LolC recruits LolA via its two structure-specific features, a β-hairpin “Hook” and a three-residue “Pad” (22), while LolE was identified by in vivo cross-linking as the lipoprotein binding site (21). Recent cryogenic electron microscopy (cryo-EM) structures of *E. coli* LolCDE revealed how lipoproteins are recognized (23, 24). The central channel formed by LolC and LolE transmembrane helices accommodate the three acyl chains of the lipoprotein in two hydrophobic pockets elevated above the membrane plane while, in agreement with the cross-linking data, the peptide portion extends upwards, forming interactions with LolE but not LolC. In some species such as *Neisseria* and *Francisella*, LolC and LolE are replaced by a single protein, LolF, which presumably carries both functions (25). Lipoproteins released by LolCDE to LolA are then trafficked across the periplasm where they are passed to LolB, which catalyzes insertion into the outer membrane (Fig. 1*A*). LolA and LolB share structural homology, both being incomplete β-barrels with a central hydrophobic cavity closed by an alpha helical lid (26), but perform distinct functions. LolB does not interact with LolC due, at least in part, to the absence of a C-terminal extension that in LolA binds the Pad of LolC (22). Conversely, LolA cannot release lipoproteins into membranes (27, 28). Transfer of lipoproteins between LolA and LolB is not completely understood but in vivo cross-linking and NMR data suggest that it occurs with the two cavities of the proteins facing each other, in a “mouth-to-mouth” model (20, 29).

Here, we present the crystal structure of LolA in complex with a lipoprotein, providing a molecular description of the interaction and a rationale for the capacity of the chaperone protein to transport evolutionarily divergent lipoproteins. Biochemical, biophysical, and further structural data illustrate that lipoprotein loading is a carefully orchestrated process where aberrant loading is possible but prevents further trafficking. Our data deliver structural insights into a key lipoprotein trafficking intermediate, which may support the development of therapeutic strategies targeting the Lol system and provide a significant step toward the full understanding of a process fundamental to bacterial physiology.

## Results

### Trapping a LolA–Lipoprotein Intermediate Suitable for Structural Investigation.

LolA–lipoprotein complexes can be isolated from the periplasm after overexpression of the two proteins (28) but these complexes were refractory to crystallization. As illustrated in Fig. 1*B*, outer membrane lipoproteins only share a suite of four conserved residues forming the lipobox, while the N-terminal sequence immediately following the triacylated cysteine is highly variable. In most lipoproteins, this region corresponds to a flexible linker joining the lipoprotein triacyl group to the globular domain (14), which we reasoned could prevent crystallization. Thus, to capture a LolA–lipoprotein intermediate favorable for structural studies, we introduced a tobacco etch virus (TEV) protease cleavage site in the N-terminal region of Pal to permit targeted removal of the lipoprotein linker and globular domain (Fig. 1*C*). Introduction of the protease cleavage site did not affect lipoprotein maturation or Lol trafficking of the modified full-length (FL) Pal, hereafter Pal_TEV/FL_, to the outer membrane in vivo (Fig. 1*D*). Furthermore, coexpression of LolA with either wild-type Pal or Pal_TEV/FL_ and isolation of the resultant complexes show that the two lipoproteins associate equally well with the chaperone (Fig. 1*E*). Subsequent in vitro transfer assays demonstrate that Pal_TEV/FL_ is released to LolB as efficiently as the wild-type protein and that following protease treatment, a triacylated fragment, Pal_13_, containing only the thirteen N-terminal residues of Pal and therefore lacking the globular domain, is also successfully transferred. Taken altogether, our strategy results in a physiologically relevant intermediate of the lipoprotein trafficking pathway suitable for structural study.

### Crystal Structure of the LolA–Lipoprotein Complex.

**Fig. 2.**
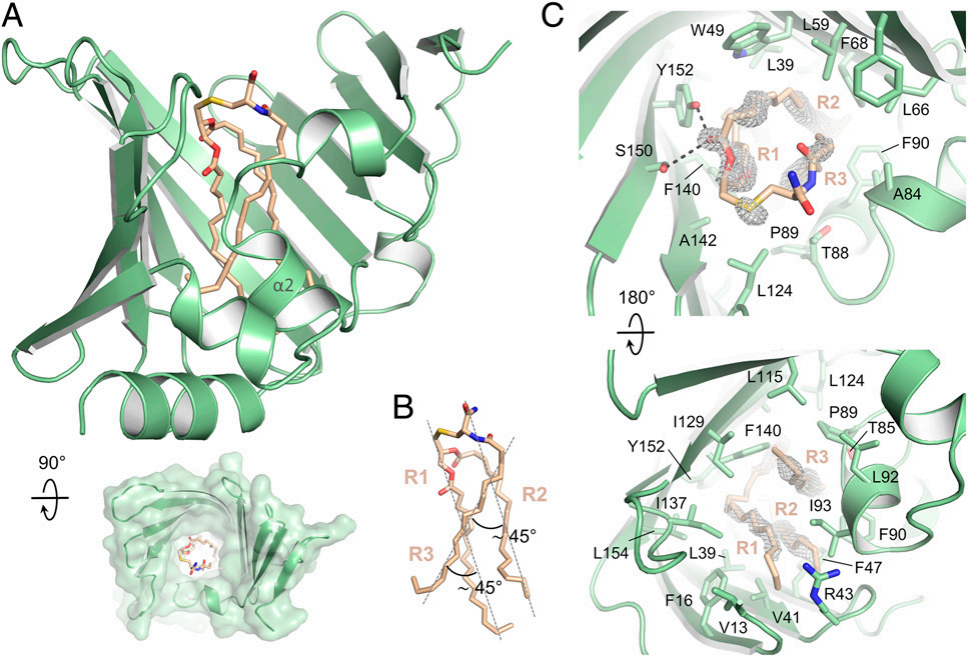
Crystal structure of a LolA–lipoprotein complex. (*A*) Overall structure of LolA (green) bound to a lipoprotein (salmon, stick representation). (*A*, *Inset*) Top view of the structure showing the entrance of the LolA cavity. (*B*) Close-up view of the lipoprotein acyl chains showing their relative orientation. (*C*) Residues of LolA interacting with the lipoprotein acyl chains viewed from the protein cavity entrance (*Top*) and from the base of the cavity (*Bottom*). The gray mesh represents the difference omit map of the lipoprotein ligand contoured at 3.0 sigma. For clarity, L10, located at the bottom of the cavity, is not shown.

We determined the crystal structure of LolA–Pal_13_ at 1.83-Å resolution. The protein complex crystallized in space group I4 with one molecule per asymmetric unit. The structure is shown in Fig. 2*A*, with X-ray data and refinement statistics listed in *SI Appendix*, Table S1. Electron density of the protein model is presented in Movie S1 together with the ligand omit difference and polder maps (30). We resolved density for the triacylated cysteine but not for the rest of the peptide chain, suggesting that LolA does not make specific interactions with the lipoprotein amino acid chain, consistent with our demonstration that transfer to LolB can occur in the absence of the lipoprotein globular domain (Fig. 1*E*).

The three acyl chains of the ligand are inserted deeply in the U-shaped cavity of LolA with electron density compatible with 16-carbon chains, consistent with the predominant species in *E. coli* lipoproteins (31, 32). The acyl chains pack together with R1 and R2 running straight down and R3 packed against the other two chains at ∼45° angle (Fig. 2*B*). The lipoprotein triacyl group is stabilized by van der Waals (VDW) interactions with multiple residues forming the hydrophobic cavity of LolA (Fig. 2*C*). From the top to the bottom of the cavity: F68, L59, L66, A84, L124, S150, W49, A142, Y152, T85, T88, F47, L39, F140, P89, F90, L115, L154, I129, V41, L92, I93, I137, F16, R43, L10, and V13 all participate in the stabilization of the lipoprotein acyl chains. Interestingly, two polar interactions between the carbonyl group of the R2 ester linkage and the side chains of both S150 and Y152 also stabilize the lipoprotein.

**Fig. 3.**
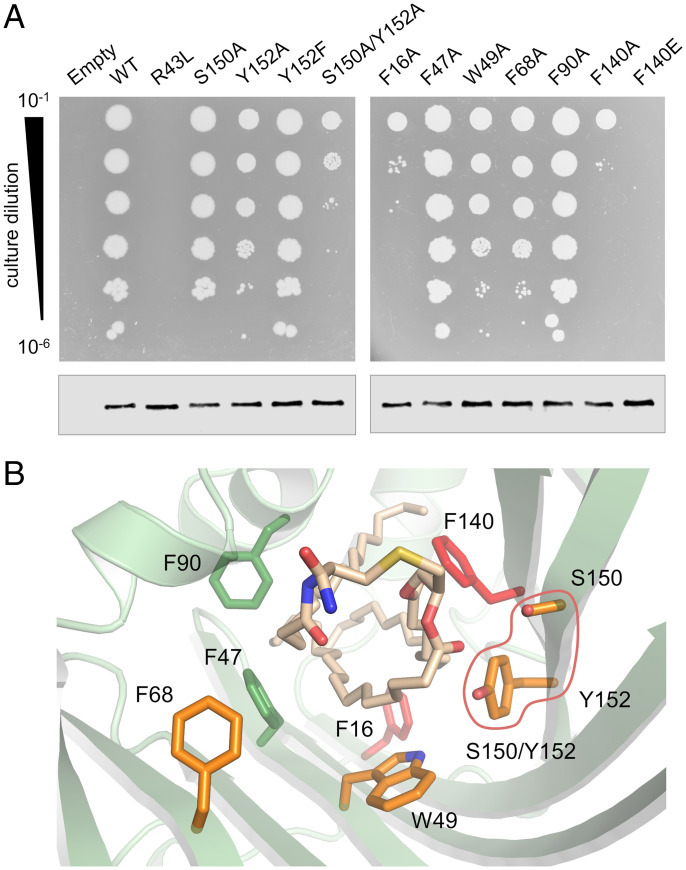
Probing the importance of LolA residues involved in lipoprotein interaction. (*A*, *Top*) Ability of LolA variants to support bacterial growth. Cultures of a conditional *lolA* knockout strain, BW49, carrying empty vector, plasmid-borne *lolA*, or indicated variant were serially diluted on plates lacking the inducer required for expression of chromosomal, wild-type *lolA*. (*A*, *Bottom*) Immunoblot showing expression level of plasmid-borne wild-type *lolA* or indicated variant in BW49 cells supported by growth of chromosomal *lolA*. (*B*) Location of the mutated residues in the LolA–lipoprotein structure. Colors indicate the impact of specific residue substitutions on LolA function: wild-type–like, green; modestly impaired, orange; strongly impaired, red. The outline denotes the result obtained with the simultaneous mutation of S150 and Y152.

Expression of LolA is essential, so to probe the importance of residues underpinning these interactions in vivo, we assayed the ability of LolA variants to support growth in a conditional knockout strain (Fig. 3 *A*, *Top*). In the absence of chromosomal *lolA* expression, plasmid-borne *lolA* supports growth, whereas empty vector or the defective R43L mutant do not, consistent with previous results (33). We first investigated the importance of the residues involved in polar interactions with the triacyl group by removing their hydrogen bonding ability. Alanine substitution of S150, or replacement of Y152 by alanine or phenylalanine, have a minimal impact on growth, while the simultaneous mutation of both residues had a substantial effect, suggesting that conservation of hydrogen bonding capacity in that region is important for LolA function.

We next investigated the importance of individual hydrophobic interactions by targeting the aromatic residues surrounding the acyl chains (Fig. 2*C*). Alanine substitution of F47, W49, F68, and F90 either did not affect or had little effect on growth. Conversely, F16A and F140A mutations had a significant impact and introduction of a charged, glutamate residue at position 140 in the center of the cavity led to complete growth inhibition. Failure was not due to absence of expression since these variants were expressed at a similar level as the wild-type protein when growth was supported by the expression of chromosomal *lolA* (Fig. 3 *A*, *Bottom*). Mapping these data onto the structure reveals that S150, Y152, F16, and F140 are all located on the same face of the cavity (Fig. 3*B*), suggesting that this side of the LolA β-barrel plays a dominant role in LolA function.

### The LolA–Lipoprotein Complex Is Disrupted by MAC13243 Inhibitor In Vitro.

**Fig. 4.**
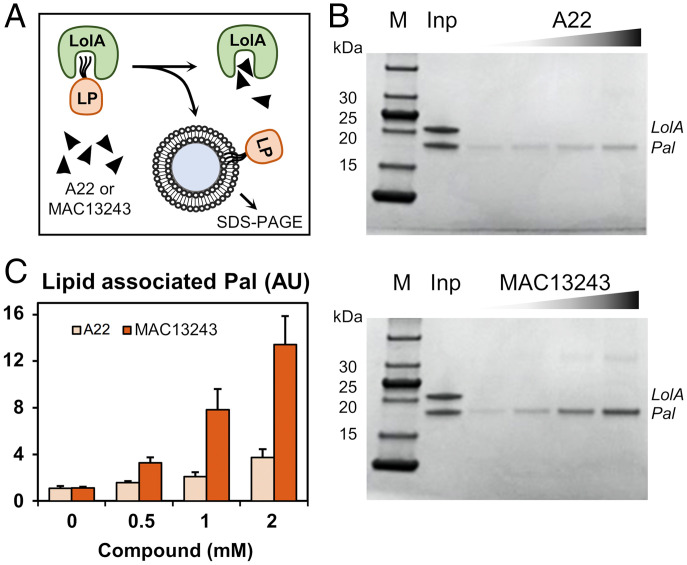
A22 and MAC13243 inhibitors dissociate bound lipoprotein from LolA. (*A*) Schematic representation of LolA–Pal dissociation by A22 or MAC13243 in the presence of *E. coli* phospholipid-coated beads. (*B*) LolA–Pal complex was incubated for 30 min with 0, 0.5, 1, or 2 mM of A22 (*Top*) or MAC13243 (*Bottom*) in the presence of the phospholipid-coated beads. After several washes, the lipid-coated beads were collected, and associated Pal was analyzed by SDS-PAGE. Purified Lol–Pal complex (Inp, input) was included as a control and molecular masses of protein standards (M) are indicated. (*C*) Quantification of Pal associated with lipid-coated beads determined as mean ± SD arbitrary units (AU) for triplicate determinations.

We then challenged the LolA–lipoprotein interaction with MAC13243, a small antibacterial compound whose activity could be abrogated by overexpression of LolA (34, 35) and which was proposed, on the basis of MD simulations, to disrupt the chaperone–lipoprotein complex (36). To investigate whether MAC13243, and its small structural analog A22, indeed target the interaction of LolA with lipoprotein, we examined the ability of both compounds to disrupt the LolA–Pal complex by measuring Pal release and subsequent insertion into phospholipid-coated beads (37) (Fig. 4*A*). We found that both molecules dissociate Pal from LolA in a concentration-dependent manner (Fig. 4 *B* and *C*), indicating that lipoprotein-bound LolA is a direct target of the antibacterial compounds. In line with previously reported bacterial susceptibility and LolA affinities (34, 35), we observed a greater effect of MAC13243 over A22 at identical concentration. We next questioned whether the inhibitors could also impact LolA at other stages of the lipoprotein transport cycle. We found that A22 and MAC13243 molecules do not affect LolA–LolC association (*SI Appendix*, Fig. S2 *A*–*C*) or the transfer of lipoproteins from LolA to LolB (*SI Appendix*, Fig. S2 *D*–*F*). Taken altogether, these results demonstrate that the effect of these compounds on LolA function occurs at the level of association with lipoprotein.

### Sequential Opening of the LolA Cavity.

**Fig. 5.**
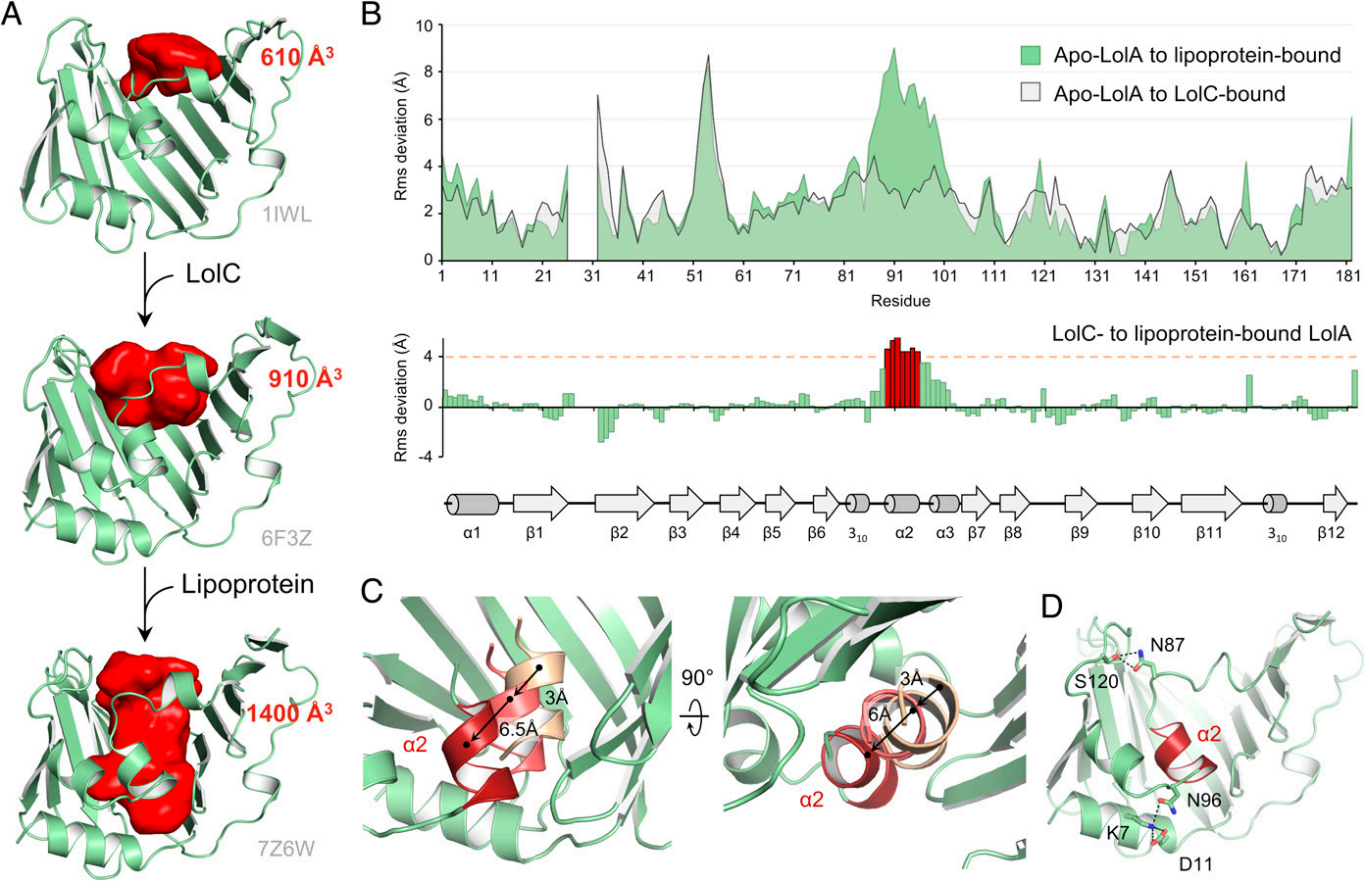
Conformational rearrangements of LolA upon LolC and lipoprotein association. (*A*) Conformational transition of LolA upon LolC interaction and lipoprotein association. The LolA cavity is visualized as a solid red “footprint” with corresponding volume indicated. (*B*) Residue-by-residue rmsd plots of free state (apo) LolA (1IWL) to LolC-bound (6F3Z, gray) or to lipoprotein-associated states (7Z6W, green). A second graph highlighting the residue deviations between LolC- and lipoprotein-bound structures is shown underneath, together with the protein secondary structure linear representation. Residues displaying deviation greater than 4 Å are in red. (*C*) Front and bottom close-up views of helix α2 upon its transition from apo LolA (1IWL, light pink), to LolC-bound (6F3Z, pink) and lipoprotein-liganded (7Z6W, red). (*D*) Interactions stabilizing helix α2 with the LolA core structure in the lipoprotein–LolA structure.

To function, LolA must transition from a LolC-bound conformation to a lipoprotein-liganded form. We previously revealed that binding of the LolC Hook primes LolA to receive lipoproteins by opening the mouth of the chaperone cavity, inducing a modest displacement of the central α2 helix (22). Here, we show that the triacyl moiety opens the deepest part of the LolA pocket by a further 1.5-fold, increasing the cavity volume to ∼1,400 Å^3^ (Fig. 5*A*). We generated a molecular morph highlighting the presumed motions undergone by LolA through its sequential transition from an apo form to LolC-bound and lipoprotein-associated states (Movie S2). Structural alignment and calculation of rmsd reveal that region 89 to 95 exhibits considerable displacement (>4 Å) upon lipoprotein association once the protein is in a LolC-bound conformation, whereas the rest of the protein does not (Fig. 5*B*). This region corresponds to the central α2 helix of the LolA lid that is pushed significantly both toward the bottom of the cavity and away from the protein half β-barrel (Fig. 5*C*). Relative to apo LolA, the helix is displaced by about 9 Å, a motion required for the accommodation of the three acyl chains deep inside the cavity. Interestingly, helices α2 and α3 of LolA lid conserve their relative position along this conformational transition by a salt bridge between R95 and D100 (*SI Appendix*, Fig. S3*A*). In the lipoprotein-bound structure, helix α2 is also stabilized by hydrogen bonds with K7 and S120 (Fig. 5*D*).

A key arginine residue, R43, maintains the LolA helical lid in a closed form in the apo structure through hydrogen bonds with the I93, A94, and L10 main chains (38). In subsequent states of LolA, we found that, upon LolC association, R43 maintains interaction with lid helix α2 as they both move closer to V13 at the base of the cavity and releases the lid upon lipoprotein transfer to fully open the cavity allowing deep insertion of the lipid chains (*SI Appendix*, Fig. S3*B* and Movie S4).

### Crystal Structure of R43L LolA–Lipoprotein Complex Reveals an Altered Ligand Binding Mode.

**Fig. 6.**
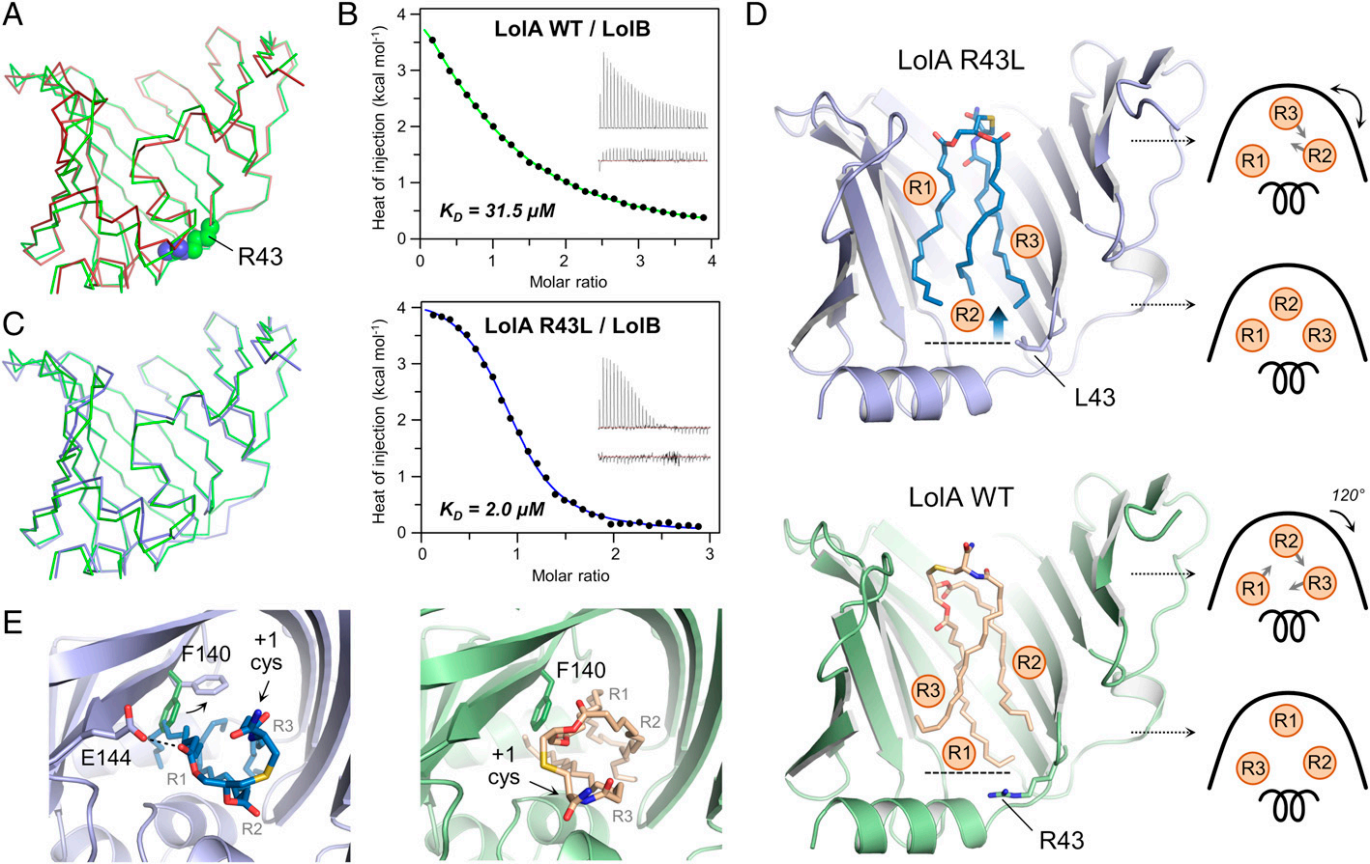
Structural and biophysical analysis of R43L LolA, a variant incapable of lipoprotein transfer to LolB. (*A*) Structural alignment of LolA lipoprotein (green) and R43L LolA in its open state (2ZPD, red). (*B*) Calorimetry isotherms showing interaction between LolB and wild-type LolA or R43L with dissociation constants *K_D_* indicated. The main figure represents background-corrected heats of injection with a fitted binding curve, while the *Inset* shows two thermograms corresponding to injection of the indicated LolA protein into cell-contained LolB (*Top*) or buffer (*Bottom*). (*C*) Superposition of LolA–lipoprotein (green) and R43L LolA–lipoprotein (blue) structures. (*D*) Crystal structure of R43L LolA–lipoprotein complex and comparison with the corresponding structure obtained with wild-type LolA. A schematic representation of lipoprotein acyl chain projection in wild-type LolA (WT) and R43L cavities is shown on the *Right* of each structure. (*E*) Close-up view of the cavity entrance in LolA R43L– (*Left*) and wild-type–lipoprotein structures (*Right*), highlighting the rotation of the F140 side chain.

The importance of R43 in the lipoprotein transfer process was underlined by the identification of a mutant at this position, R43L, that though competent to accept lipoproteins from LolCDE was unable to transfer them to LolB (33). The leucine substitution disrupts the interaction between helix α1 and the lid helix α2, resulting in the mutant adopting an open cavity relative to the wild-type protein (38, 39). Alignment with our lipoprotein-bound LolA structure unveiled the similarity in conformation of the two proteins (rmsd of 0.82 Å over 165 residues) (Fig. 6*A*), indicating that the open R43L mutant mimics the LolA–lipoprotein-associated state. Interestingly, we found that LolB displays a 10-fold greater affinity for the R43L protein than for wild-type LolA (Fig. 6*B* and *SI Appendix*, Table S2), showing that LolB is able to discriminate the two conformations of LolA and that its deficiency to receive lipoproteins is not due to a lack of interaction with R43L LolA. We reasoned that the inability to transfer lipoproteins could be due to differences in the ligand-induced conformation of the R43L variant relative to the wild-type protein or to an alternative lipoprotein binding mode. To resolve these possibilities, we produced the LolA R43L–lipoprotein complex and determined its crystal structure. Corresponding X-ray collection and refinement data are given in *SI Appendix*, Table S1 with ligand omit maps shown in Movie S3. The protein conformation is similar to that of liganded WT LolA (Fig. 6*C*) with an rmsd of 0.68 Å over 167 residues, but the three acyl chains of Pal are positioned differently in the protein cavity (Fig. 6*D* and *SI Appendix*, Table S3). Here, the R1 and R3 chains run almost parallel with R2 inserting in the middle at the cavity base. The R1 chain is less deeply inserted in the R43L protein cavity by about 4 Å compared to its position in the wild-type structure. The cysteine carbonyl is rotated by about 180°, pointing toward the β-barrel curve in the R43L complex while facing the helical lid in the wild-type LolA structure (Fig. 6*E*). In addition to the engineered R43L mutation, there are two other key differences in the mutant structure. First, the F140 side chain is flipped toward the β-barrel curve of the protein, in a position that conflicts with the location of chain R1 observed in the wild-type complex structure (Fig. 6*E*). Second, the shallower insertion of the ligand means that Y152 is unable to interact with the R2 acyl chain carbonyl as it does in the wild-type structure. Instead, E144 situated nearer the mouth of the cavity forms a hydrogen bond with the carbonyl backbone of acyl chain R1.

To rationalize why this mutant cannot transfer lipoproteins to LolB, we performed protein–protein docking using open LolA from our LolA–lipoprotein complex and LolB, resulting in a model in good agreement with previous in vivo cross-linking data (*SI Appendix*, Fig. S4*A*) (20), and positioned both lipoprotein conformations in this LolA–LolB complex. We found that the Pal +1 cysteine observed in the LolA R43L structure sterically clashes with LolB, while in the LolA wild-type structure, it is accommodated in a LolA surface groove allowing the linker joining the triacyl group to the lipoprotein mature domain to exit the complex (*SI Appendix*, Fig. S4*B*). It is therefore likely that the aberrant lipoprotein conformation prevents productive association with LolB, explaining why lipoproteins cannot be released from R43L LolA.

We next considered why the R43L mutation results in an altered lipoprotein binding mode and reasoned that the ability of R43L LolA to adopt a fully open state modifies its association with LolC. To challenge this hypothesis, we used the fluorescently labeled lipid, DAUDA, to probe complexes of periplasmic LolC with wild-type LolA or the R43L variant. The emission fluorescence spectra indicate that, under the same conditions, more lipid probe associates when LolC is bound to LolA R43L versus the wild-type protein (*SI Appendix*, Fig. S4*C*). Differences in binding were also observed by isothermal titration calorimetry (ITC) where titration of LolC and wild-type LolA generates a typical, sigmoidal response, while association of LolC and LolA R43L resulted in biphasic isotherms (*SI Appendix*, Fig. S4*D* and Table S2). Altogether, these experiments suggest that the R43L mutation perturbs the association with LolC, resulting in an abortive LolA–lipoprotein complex, incompetent for LolB binding and/or lipoprotein transfer. The data further underline that the LolC-mediated sequential opening of LolA is a tightly controlled process, crucial for inserting lipoprotein in a manner productive for onward transfer to LolB.

### Lipoprotein Transfer from LolCDE to LolA.

LolA is recruited to LolCDE by two distinct interactions with the LolC periplasmic domain where the LolA cavity and C terminus engage the LolC Hook and Pad, respectively (22). Superposition of our triacyl-bound LolA with either the LolA–LolC crystal structure (22) or the cryo-EM LolA–LolCDE complex (23) reveals a steric clash between the LolC Hook and the lipoprotein triacyl group inside the LolA cavity (*SI Appendix*, Fig. S5*A*). Insertion of the lipoprotein acyl chains therefore physically disengages the LolC Hook, thereby initiating release of LolA from the transporter. The relative orientation of the lipoprotein triacyl moiety, LolA and LolCDE, indicates that the acyl chains insert into the LolE-facing side of LolA cavity by sliding between the LolC Hook and the residues belonging to the β10, β11 strands and the loop joining β8 and β9 of LolA (*SI Appendix*, Fig. S5*B*). This face of the chaperone includes the phenylalanine residue, F140, highlighted as important for LolA function (Fig. 3*B*). We predict that transfer is initiated by a single acyl chain as the volume between the LolC Hook and the opposite edge of the LolA β-barrel does not allow the simultaneous transfer of multiple acyl chains (*SI Appendix*, Fig. S5*C*).

## Discussion

Outer membrane–destined lipoproteins are trafficked through the aqueous periplasmic environment by forming a soluble complex with the chaperone protein LolA. We generated LolA–Pal lipoprotein complexes amenable to crystallization (Fig. 1) and solved the crystal structure of the complex, revealing all three lipoprotein acyl chains are bound deep inside the protein cavity in a precise conformation (Fig. 2). Multiple LolA residues interact with the acyl chains, but structurally targeted mutagenesis demonstrated the importance of specific aromatics as well as a pair of polar amino acids (Fig. 3). These residues, located on one face of the LolA cavity, do not form interactions with the LolC Hook (22) and are therefore presumably important for lipoprotein transfer events.

LolA does not distinguish inner and outer membrane lipoproteins; sorting occurs exclusively at the level of LolCDE as single mutations in the transporter are sufficient to abrogate selectivity (40, 41). Our structure demonstrates the lipoprotein amino acids do not interact with LolA, explaining not only this lack of selectivity but, more importantly, how LolA is able to transport lipoproteins diverse in structure and sequence. Interaction is solely mediated by the lipoprotein triacyl moiety binding inside the protein cavity. A previous study reported that disruption of a hydrophobic patch at the surface of *Pseudomonas* LolA impaired protein function, suggesting some of the lipoprotein acyl chains could bind outside of the cavity (42). Consideration of the LolA–LolC structure (22) suggests that the mutations, located close to the LolA C terminus, are likely to perturb the interaction of LolC and LolA rather than affecting the acyl chain binding. Our study unequivocally demonstrates accommodation of all acyl chains within the hydrophobic cavity, and it is therefore reasonable to infer that the structure presented here reflects the binding mode of all outer membrane–destined lipoproteins.

Small molecule inhibitors able to bind LolA in vitro (34, 35), but also targeting MreB (43), the bacterial actin homolog, have been reported. We challenged our LolA–lipoprotein complex with MAC13243 and A22 molecules and found that both disrupt the interaction in a concentration-dependent manner (Fig. 4). The presumed affinity of these compounds is modest, indicating that they are unlikely to play a decisive role in the inhibition of lipoprotein trafficking in vivo. However, the work presented here has the potential to screen variant small molecules that can specifically target LolA–lipoprotein association.

Our data complete the repertoire of the conformational changes adopted by LolA during lipoprotein trafficking, revealing the sequential opening of the cavity from an unliganded conformation to a LolC- and finally lipoprotein-bound state (Fig. 5). Structural resolution of a LolA R43L–lipoprotein complex revealed that acyl chains are inserted in an aberrant position incompatible with further trafficking (Fig. 6). The ability of this variant to adopt a prematurely open state most likely modifies its association with LolC, underscoring the importance of the LolCDE-orchestrated opening of wild-type LolA to accommodate the triacyl moiety in a defined position, productive for transfer to LolB.

**Fig. 7.**
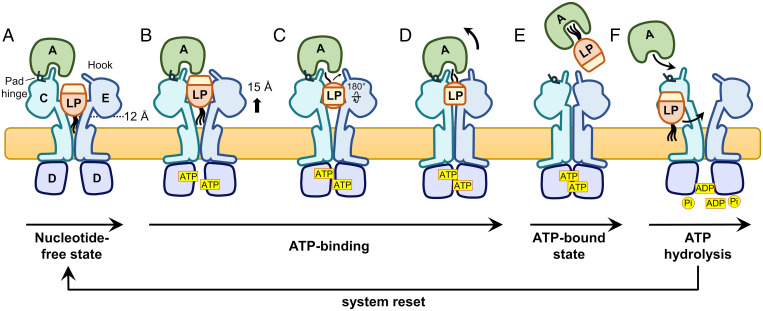
Model of lipoprotein transfer from LolCDE to LolA. (*A*) Lipoprotein (LP) and LolA bind LolCDE in the open, nucleotide-free state. In this conformation, the LolE Hook is in a horizontal position, capping the transporter, while the LolC Hook is engaged in association with LolA. (*B*) Binding of ATP induces the closure of the transporter transmembrane helices first, which elevates the lipoprotein in a cavity formed by the LolC and LolE periplasmic domains. (*C*) The continuing closure of the transporter forces the acyl chains to rotate in this cavity by 180° clockwise around an axis running from LolC to LolE. Rotation of the triacyl moiety in the other direction would be prevented by the lipoprotein mature domain that protrudes from LolCDE. The Hook of LolE relocates to bridge the gap between LolA and LolE. (*D*) The first lipoprotein acyl chain engages the LolA cavity, slightly displacing LolA from the LolC Hook and inducing a “rocking” movement of the chaperone where the interaction with LolC Pad acts as a hinge. (*E*) The second and third chains engage LolA, dislodging the chaperone–lipoprotein complex from the transporter. (*F*) Hydrolysis of ATP resets the system.

A combination of our data and published structures of LolC–LolA (22) and LolCDE (23, 24), allows us to propose a scheme for lipoprotein transfer from LolCDE to LolA. Lipoproteins and LolA are recruited by LolCDE without energy input, binding to the open, nucleotide-free state of the transporter (Fig. 7*A*). Following the mechanotransmission mechanism described for type VII ABC transporters (44), ATP binding to LolCDE first induces the dimerization of the nucleotide-binding domains, leading to the closure of LolC and LolE transmembrane helices. This movement forces the elevation of the bound acyl chains by about 15 Å in a “translocation” step toward a cavity formed between the periplasmic domains of the two proteins (Fig. 7*B*).

Further studies are required to define the precise path followed by the acyl chains in LolCDE yet our LolA–lipoprotein structure demonstrates that the triacyl moiety orientation must invert relative to its position in LolCDE-bound state, which is likely to occur within this cavity driven by the convergence of the LolC and LolE periplasmic domains. As indicated by our structural analysis, a single chain is likely to initiate the transfer into LolA and, among the three acyl chains, R3 would have the shortest route to reach the chaperone during this rotation of the triacyl moiety and thus may be in prime position to engage the LolA cavity. In the LolA–LolCDE structure (23), the Hook of LolE caps the transporter, preventing lipoprotein access to the chaperone, and its movement toward LolA would not only open passage to the chaperone cavity but also exclude solvent access between the two proteins to permit acyl chain transfer (Fig. 7*C*). The insertion of the first acyl chain, plausibly R3, initiates disengagement of the LolC Hook from the LolA cavity, which rocks away from LolE, by pivoting on a hinge created by the LolA–LolC Pad interaction (Fig. 7*D*). Insertion of the subsequent acyl chains pushes LolA further away from LolC, detaching the chaperone protein from the transporter, possibly aided by conformational changes within the periplasmic domain of LolC that disrupt the LolA–LolC Pad interaction (Fig. 7*E*). Finally, ATP hydrolysis resets the system, allowing the entry of another lipoprotein in LolCDE transporter and recruitment of LolA (Fig. 7*F*).

Our model suggests an important role for the R3 acyl chain in the transport process supported by the essentiality in wild-type *E. coli* of Lnt, the enzyme responsible for its transfer to lipoproteins (45, 46). In other bacteria, including pathogenic *Neisseria*, *Francisella*, and *Acinetobacter* species, Lnt may be absent or its deletion better tolerated (25, 47, 48). LolA proteins in these organisms are not predicted to be different in structure (49), it will be interesting to see in these cases how the conformational changes in LolA differ to accommodate the smaller volume of the diacyl moiety.

The structure presented here demonstrates that all three lipoprotein acyl chains bind within the LolA cavity so it is tempting to speculate that the structurally related outer membrane lipoprotein receptor, LolB, accommodates the lipoprotein in a similar manner. More studies are required to detail LolB association with the triacyl moiety and how this interaction permits the subsequent release of lipoprotein into the outer membrane. In summary, the structure of the LolA–lipoprotein complex presented here reveals the molecular details of an intermediate at the heart of the lipoprotein trafficking system, providing a crucial step toward a complete understanding of a fundamental transport process.

## Materials and Methods

Complete methods are available in *SI Appendix*, *SI Methods*.

In brief, complexes of LolA and Pal_WT_, Pal_TEV/FL_, or Pal_13_ were generated by overexpressing, in *E. coli* C43 (DE3) cells, strep-tagged LolA, and his-tagged Pal containing, when indicated, an internal TEV cleavage site. Cells were converted into spheroplasts and proteins recovered from the periplasmic fraction before purification by immobilized metal affinity chromatography (IMAC). Transfer of Pal_WT_, Pal_TEV/FL_, or Pal_13_ from LolA to LolB was assessed by a pull-down assay using microbatch spin columns. His-tagged soluble mLolB was incubated at a 2:1 ratio with tag-free LolA–Pal complexes for 30 min before loading the mixtures onto Ni-resin. Bound proteins were washed three times, eluted, and analyzed by sodium dodecyl sulfate-polyacrylamide gel electrophoresis (SDS-PAGE). Localization of Pal constructs was performed by converting induced and uninduced *E. coli* cultures into spheroplasts and recovering outer membranes by ultracentrifugation. Pal proteins were detecting by immunoblotting using anti-His antibodies.

A *lolA* conditional knockout strain, BW49, was created by inserting the *lolA* allele under the control of an arabinose promoter at the lambda attachment site and then removing the native gene by lambda red recombination (50). This strain was then used to monitor the ability of plasmid-borne variants of LolA to support growth in the absence of arabinose. LolA–Pal_13_ was prepared by TEV cleavage of LolA–Pal_TEV/FL_ protein and crystallized by the sitting-drop vapor-diffusion method at 11 mg/mL in a solution containing 2.1 M DL-malic acid, pH 6.0. Crystals were cryoprotected with the reservoir solution supplemented with 20% glycerol and diffracted remotely at Diamond synchrotron. The structure was solved using the CCP4 suite (51). X-ray data were processed with Imosflm (52), scaled with Aimless (53), and solved by molecular replacement with Phaser (54) using LolA (Protein Data Bank (PDB) accession code 1UA8) as the search probe. Model building and refinement used Coot (55) and Refmac (56). The final structure was validated with Rampage (57) and Procheck (58). Complexes of LolA R43L–Pal_28_ were generated as described for LolA–Pal_13_, concentrated to 6.5 mg/mL and crystallized in 30% PEG3350, 50 mM bis-Tris, pH 6.0, in the presence of seeds. Crystals were diffracted on X06S at the Swiss Light Source (SLS). LolA–Pal complex was challenged with A22 and MAC13243 compounds in the presence of *E. coli* phospholipid bilayer-coated beads, prepared as previously described (37). Proteins were incubated with 0, 0.5, 1, or 2 mM of each compound for 30 min, washed three times with buffer before collecting the lipid-coated beads by centrifugation, and eluting them in SDS and urea. Bound proteins were analyzed by SDS-PAGE. ITC experiments were performed in a VP-ITC calorimeter (Malvern Panalytical) at 25 °C, with a stirring speed of 300 rpm. LolA wild-type or R43L protein (250 or 500 µM) was injected into the cell containing mLolB (25 or 30 µM). For each titration, a control experiment was performed by injecting LolA into buffer. The resulting data were analyzed with the PEAQ-ITC software package (Malvern Panalytical).

## Supplementary Material

Supplementary File

Supplementary File

Supplementary File

Supplementary File

Supplementary File

## Data Availability

Coordinates and structure factor data have been deposited in the PDB (accession no. 7Z6W (59) 7Z6X (60)).
