# Supplementary material for: Structural basis of lipoprotein recognition by the bacterial Lol trafficking chaperone LolA
Source: Proc Natl Acad Sci U S A. 2022 Aug 29;119(36):e2208662119. doi: 10.1073/pnas.2208662119 (PMC9457489; doi:10.1073/pnas.2208662119)
Supplement: Supplementary File [file pnas.2208662119.sapp.pdf]

Supplementary Information for

Structural basis of lipoprotein recognition by the bacterial Lol trafficking chaperone LolA

Elise Kaplan[1], Nicholas P. Greene*[1], Abigail E. Jepson, Vassilis Koronakis*

Department of Pathology, University of Cambridge, Cambridge CB2 1QP, United Kingdom

*Corresponding authors: npg22@cam.ac.uk and vk103@cam.ac.uk

[1] E.K. and N.P.G. contributed equally to this work

This PDF file includes:

Figures S1 to S5
Tables S1 to S5
Legends for Movies S1 to S4
Supplementary methods
SI References

Other supplementary materials for this manuscript include the following:

Movies S1 to S4

Supplementary Figures

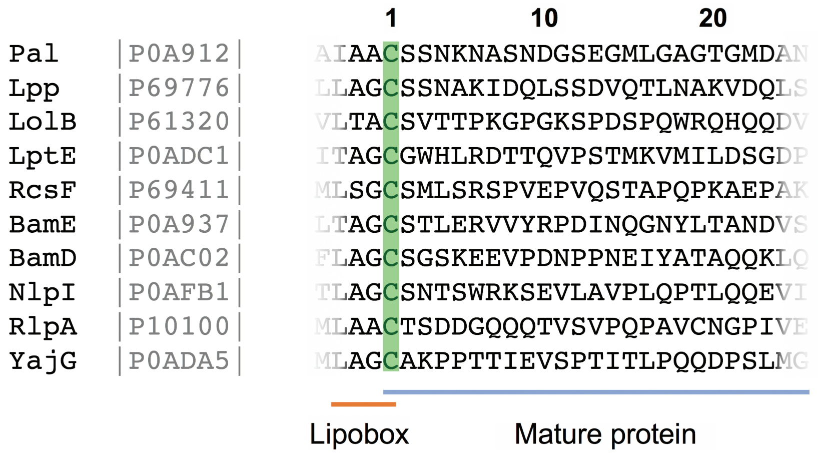

Figure S1. Sequences of the N-terminal region of selected *E. coli* outer membrane lipoproteins. Protein sequences are aligned on the invariant cysteine (highlighted in green) which constitutes the first residue of the lipoprotein after maturation by the three inner-membrane proteins Lgt, Lsp and Lnt (1). Uniprot entry codes are displayed in gray. The corresponding conservation plot is presented in **Figure 1*B***.

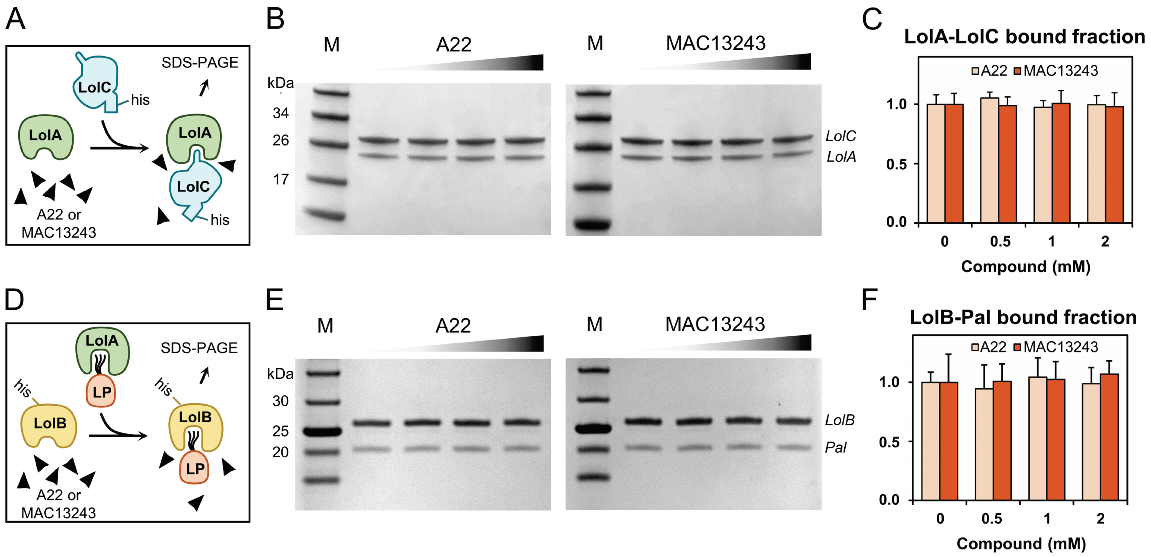

Figure S2. A22 and MAC13243 inhibitors do not affect LolA-LolC interaction nor LolA to LolB lipoprotein transfer. (*A, D*) Schematic representation of LolA-LolC interaction and lipoprotein transfer from LolA to LolB, respectively, in the presence of A22 or MAC13243 compounds. (*B*) Tag-free LolA was incubated with 0, 0.5, 1 or 2 mM A22 (*left*) or MAC13243 (*right*) before addition of His-tagged LolC periplasmic domain and loading the mixture on nickel resin. After several washes, bound proteins were eluted with imidazole and analyzed by SDS-PAGE. Molecular masses of protein standards (M) are indicated. (*C*) Quantification of LolA associated with the LolC periplasmic domain in the presence of A22 (light pink) or MAC13243 (orange). Results correspond to the ratio of LolA to LolC normalized against the value obtained without inhibitor and represented as mean ± standard deviation for triplicate determinations. (*E*) His-tagged mLolB was incubated with 0, 0.5, 1 or 2 mM A22 (*left*) or MAC13243 (*right*) before adding tag-free LolA-Pal complex and loading the mixture on nickel resin. After several washes, bound proteins are eluted with imidazole and analyzed by SDS-PAGE. Molecular masses of protein standards (M) are indicated. (*F*) Quantification of Pal associated with mLolB in the presence of A22 (light pink) or MAC13243 (orange). Results correspond to the ratio of Pal to mLolB normalized against the value obtained without inhibitor and shown as mean ± standard deviation for triplicate determinations.

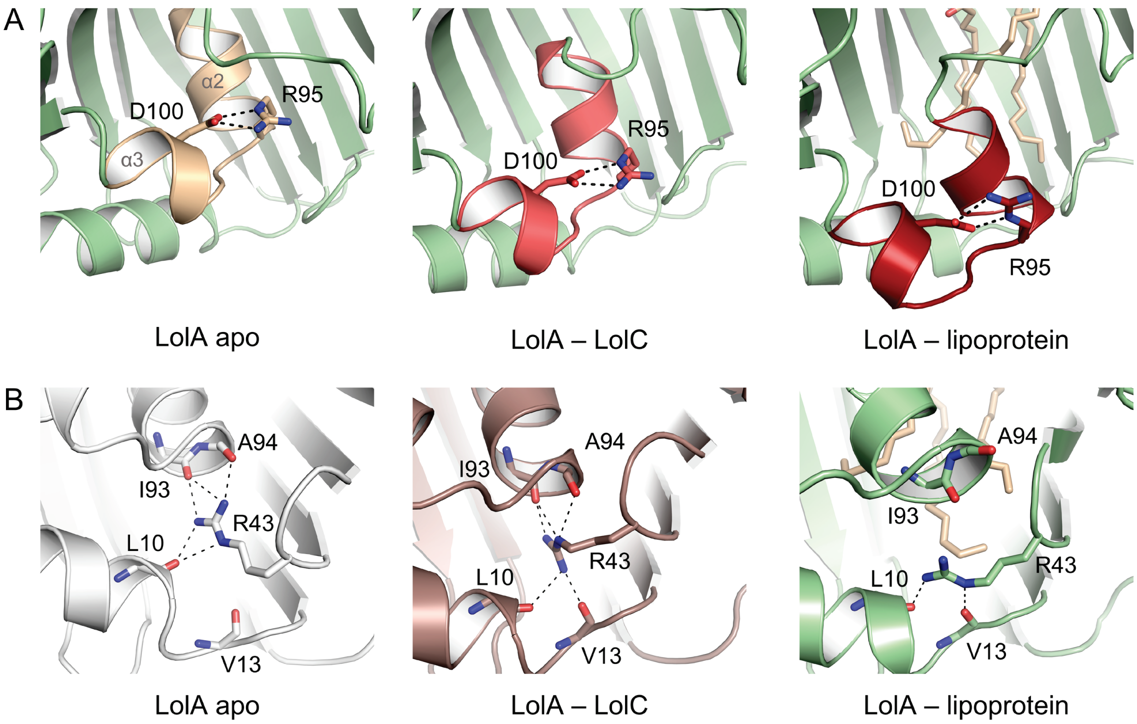

Figure S3. LolA helical lid stabilizing network in the apo, LolC- and lipoprotein-bound structures. (*A*) Salt bridge stabilizing helices α2 and α3 in apo (1IWL), LolC-bound (6F3Z) and lipoprotein-associated (7Z6W) LolA. (*B*). Hydrogen-bond network of R43 in the LolA apo structure (1IWL), the LolA-LolC complex (6F3Z) and the lipoprotein-associated state (7Z6W). A movie highlighting movement and interaction of R43L in the different LolA states is shown in **Movie S4**.

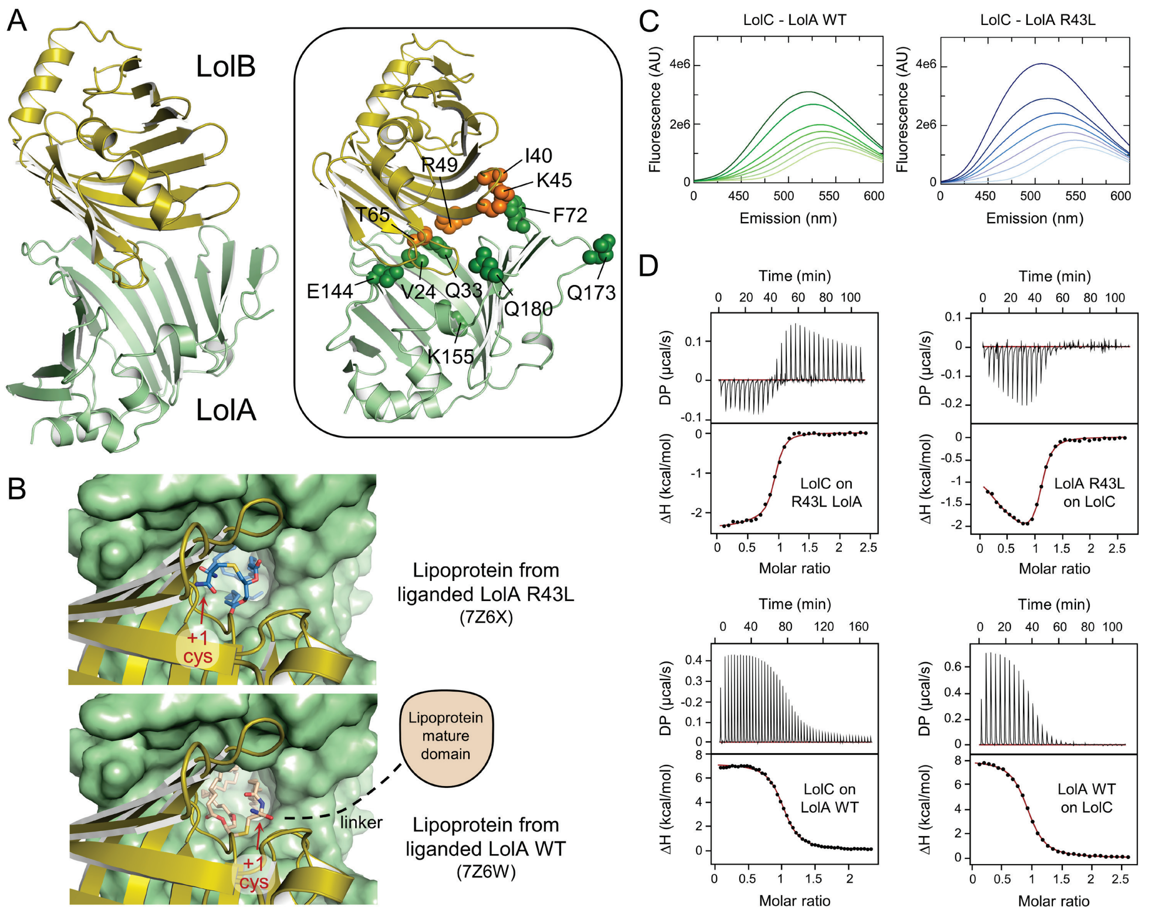

Figure S4. The LolA R43L variant displays an altered association with LolB and LolC. (*A*) Docking of mLolB (1IWM) and open LolA from the lipoprotein-bound structure (7Z6W) after removal of the ligand. *Inset*, *in vivo* photo-crosslinking data from the Tokuda lab (2) mapped on the docked structure. Residues which formed *in vivo* photo-crosslinks to LolA or LolB when mutated to pBPA are shown in orange and green, respectively. (*B*) Close-up views of the LolA cavity where the lipoprotein from the liganded LolA R43L (*Top*) or liganded LolA WT (*Bottom*) structures has been superposed onto the docking derived mLolB - LolA model. (*C*) DAUDA fluorescence spectra of increasing concentrations of LolC periplasmic domain complexed with wild-type LolA (WT) or the R43L variant. (*D*) Representative ITC profiles illustrating the association of the LolC periplasmic domain and wild-type or R43L LolA. For each titration, the thermogram is shown in the upper panel and the fitted curve of background-subtracted heats of injection in the lower panel. For the biphasic curves, values were fitted with a two-binding sites model. Values of affinities and thermodynamic parameters can be found in **Table S2**.

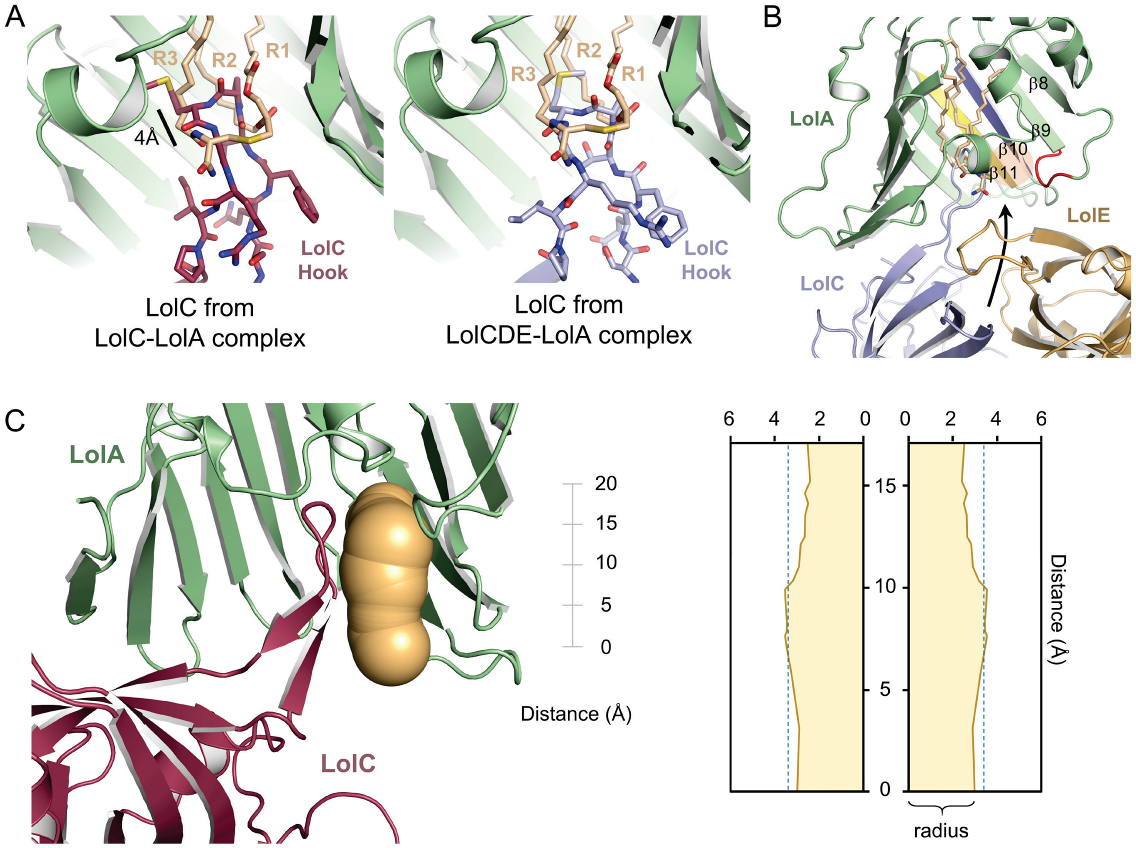

Figure S5. Insertion of lipoprotein acyl chains disengages LolA from LolCDE. (*A*) Superposition of lipoprotein-bound LolA into the LolA-LolC complex (6F3Z, *left*) and the LolA-LolCDE complex (7ARM, *right*) structures. The lipoprotein is shown in light orange and the LolC Hook in brown or blue. (*B*) Zoomed-out view displaying the path that lipoprotein acyl chains have to follow to reach LolA with β10, β11 strands and the loop joining β8 and β9 colored in blue, yellow and red, respectively. (*C*) Accessible channel between the LolC Hook and LolA β-barrel with corresponding distance plot shown on the *right*. The vertical blue line represents the van der Waals diameter of carbon (3.4 Å), only the region located outside of this line (> 3.4 Å) would allow the simultaneous transfer of two proximal acyl chains. Analysis was performed with the MOLE server (3) using the LolC-LolA structure (6F3Z) and mutating residues located between the LolC Hook and the LolA β-barrel into alanine.

Supplementary Tables

Table S1. X-ray data and refinement statistics.

	**LolA wild-type - lipoprotein complex**	**LolA R43L - lipoprotein complex**
**PDB code**	7Z6W	7Z6X
**Data Collection**		
Beamline	Diamond I04-1	SLS X06SA
Wavelength (Å)	0.9159	1.0000
**Crystal Parameters**		
Space Group	I4	P1 2_1 1
Unit Cell Dimensions (Å)	91.0, 91.0, 47.6	38.7, 63.3, 39.6
Unit Cell Angles (°)	90, 90, 90	90, 105, 90
Mosaic Spread (°)	0.33	0.50
**Reflection Data**		
Resolution Range (Å)	64.33-1.84 (1.88-1.84)	63.26-2.06 (2.12-2.06)
Unique Reflections	207055 (12981)	11241 (868)
R_{sym}	0.120 (1.316)	0.079 (1.142)
I/σ(I)	14.3 (2.0)	10.3 (1.9)
CC½	0.999 (0.536)	0.998 (0.741)
Completeness (%)	100.0 (100.0)	98.2 (98.8)
Multiplicity	12.1 (12.5)	6.3 (6.6)
Wilson B (Å^2)	25.7	43.5
**Refinement**		
Resolution (Å)	64.33 (1.84)	38.27 (2.06)
Number of Reflections	16184	10724
R_{work}	0.1811	0.2037
R_{free}	0.2162	0.2715
Rms (Bond Lengths) (Å)	0.011	0.006
Rms (Bond Angles) (°)	1.721	1.417
**Model Composition**		
Protein atoms	1555	1500
Waters	74	10
Other	/	/
**Model B-factors**		
Protein atoms (Å^2)	31.1	54.7
Waters (Å^2)	36.0	51.1
Other	/	/
**Ramachandran Statistics**		
Favoured (%)	96.2	96.1
Allowed (%)	3.8	3.9
Outliers (%)	0.0	0.0

Values in parentheses indicate the outer resolution bin.
Reflection data is as reported by Aimless (4).
Refinement statistics as reported by Refmac (5).
Ramachandran statistics from Rampage (6).

Table S2. ITC parameters for indicated protein-protein titration.

		K_D (µM)	*N*	Δ*G*	Δ*H*	-*T*Δ*S*
**LolA on mLolB**		19.1, 31.5<br>**25.3 ± 8.8**	0.81, 0.91<br>**0.86 ± 0.07**	-6.5, -6.1<br>**-6.3 ± 0.2**	5.7, 8.2<br>**7.0 ± 1.7**	-12.2, -14.3<br>**-13.3 ± 1.5**
**LolA R43L on mLolB**		2.60, 1.99<br>**2.30 ± 0.43**	1.01, 0.96<br>**0.98 ± 0.04**	-7.6, -7.8<br>**-7.7 ± 0.2**	4.3, 4.3<br>**4.3 ± 0.0**	-11.9, -12.1<br>**-12.0 ± 0.1**
**LolA on LolC**		0.74, 0.84, 0.62<br>**0.73 ± 0.11**	0.90, 1.01, 0.91<br>**0.94 ± 0.06**	-8.4, -8.3, -8.5<br>**-8.4 ± 0.1**	8.2, 8.6, 8.0<br>**8.3 ± 0.3**	-16.5, -16.9, -16.5<br>**-16.6 ± 0.2**
**LolC on LolA**		0.31, 0.42, 0.43, 0.47<br>**0.41 ± 0.07**	1.02, 0.92, 0.91, 1.21<br>**1.01 ± 0.14**	-8.9, -8.7, -8.7, -8.6<br>**-8.7 ± 0.1**	7.0, 7.7, 8.3, 6.2<br>**7.3 ± 0.9**	-15.9, -16.4, -17.0, -14.8<br>**-16.0 ± 0.9**
**LolA R43L on LolC**	**site 1***	~ 0.20, 0.20<br>**~ 0.20**	0.33, 0.37<br>**0.35 ± 0.03**	~ -9.1, -9.1<br>**~ -9.1**	PD	PD
	**site 2***	~ 0.40, 0.40<br>**~ 0.40**	0.75, 0.71<br>**0.73 ± 0.03**	~ -8.7, -8.7<br>**~ -8.7**	PD	PD
**LolC on LolA R43L**	**site 1***	~ 1.40, 1.40<br>**~ 1.40**	0.15, 0.21<br>**0.18 ± 0.04**	~ -8.0, -8.0<br>**~ -8.0**	PD	PD
	**site 2***	~ 0.17, 0.17<br>**~ 0.17**	0.92, 0,87<br>**0.90 ± 0.04**	~ -9.2, -9.2<br>**~ -9.2**	PD	PD

Independent repeats are listed followed by the mean ± standard deviation in bold. Values of *ΔG*, *ΔH* and *TΔS* are in kcal/mol. *T* = 25 °C.

*Biphasic curves where sites 1 and 2 correspond to the values obtained from the two-site binding model. The corresponding dissociation constants are apparent. The *ΔH* and -*TΔS* parameters obtained with LolA R43L and LolC were poorly defined (PD) by the fit.

LolC on LolA values were previously reported in Kaplan *et al*. 2018 (7).

Representative fits and thermograms are presented in **Figure 6** and **Figure S4**.

Table S3. Residues in LolA proximal to the lipoprotein ligand. Distance measurements are from the indicated LolA residue to the closest acyl chain atom in the LolA WT-lipoprotein (7Z6W) or the LolA R43L-lipoprotein (7Z6X) structures. The right-hand column represents residues which, when replaced by pBPA by the Tokuda lab, generated LolA-Pal cross-linked products *in vivo* (2).

		LolA WT - lipoprotein	LolA R43L - lipoprotein	LolA WT crosslink to Pal?
Distance to lipoprotein (Å) for residues located <4Å	L10	3.82	3.57	
	V13	3.74	-	
	L39	3.63	3.75	
	V41	3.71	3.54	
	R/L43	3.98	3.27	+
	F47	3.65	3.51	
	W49	3.63	3.39	
	L59	3.77	3.57	+
	A84	3.86	3.48	
	T85	3.95	3.86	
	T88	3.97	3.55	
	P89	3.49	3.88	
	F90	3.53	3.60	+
	L92	-	3.62	
	I93	3.59	3.20	+
	F127	-	3.51	+
	I129	3.88	-	
	I137	3.68	3.54	
	F140	3.35	3.69	
	A142	3.33	3.79	
	E144	3.50	3.21	+
	S150	2.53	-	
	Y152	3.27	3.55	
Distance to lipoprotein (Å) for residues located <5Å	F16	4.32	4.98	
	L66	4.52	4.38	
	F68	4.40	4.44	
	L92	4.50	-	
	L115	4.31	4.04	
	L124	4.32	4.36	
	I129	-	4.09	
	L154	4.16	-	

Table S4. List of primers for PCR amplification.

Primer	Description	Sequence (5' to 3')
P1	pBAD18-lolA_F	GCGCGAATTCAATAATTTTGTTTAACTTTAATAAGGAGATATAC
P2	pBAD18-lolA_R	GCGCTCTAGATTACTTACGTTGATCATCTACCGTGACGC
P3	pLDR9-araBAD-lolA_F	GCGCTCTAGACGATGCATAATGTGCCTGTC
P4	pLDR9-araBAD-lolA_R	GCGCGAGCTCAAAGGCCATCCGTCAGGATG
P5	lolA KO_F	AGCCTGGAATAGAGAGTAGAGGGAACTCCCGATCGGGAGTGAC GTAATTTGAGGAATAATGATGAGGGTTTTCCCAGTCACGAC
P6	lolA KO_R	AGTATTATCCGAAAAATCGAGCGACAGATTGCTCACTCAGGTGC CTCTACTTACGTTGATCATCTGCTTCCGGCTCGTATGTTG
P7	lolA KO screening_F	GCATCCGGCACTCTATCAACTG
P8	lolA KO screening_R	CAGAGGATCATAGAATGTAAATGC
P9	pAC80 lolA strep_F	ACGCTCTCCCTTATGCGACTCCTGCAAATCATAAAAAATTTATTT GCTTTGTGAG
P10	pAC80 lolA strep_R	GTTATTGCTCAGCGGTGGCAGCAGCGGCGGATTTGTCCTACTCA GGAGAGC
P11	lolA strep_F	GCGCTCATGAAAAAAATTGCCATCACCTGTGC
P12	lolA strep_R	GCGCGGATCCTTACTTTTCGAACTGCGGGTGGCTCCAAGAACCCT TACGTTGATCATCTACCGTGACGCCTTG
P13	lolA No Nde_F	CCAAACTTATTCAACTGGCACATGACACAACCTGATGAAAG
P14	lolA No Nde_R	CTTTCATCAGGTTGTGTCATGTGCCAGTTGAATAAGTTTGG
P15	Pal His_F	GCGCCATATGCAACTGAACAAAGTGCTGAAAGGGCTGATG
P16	Pal His_R	GCGCCTCGAGTTAGTGGTGGTGGTGGTGGTGAGAACCGTAAACC AGTACCGCACGACGGTTTTTGG
P17	Pal-thrombin-His_F	CTGGTGCCGCGCGGCAGCTCTCATCACCATCACCATCACTAAC
P18	Pal-thrombin-His_R	GCTGCCGCGCGGCACCAGAGAACCGTAAACCAGTACCGC
P19	lolA F16A_F	GGATAAAGTCAGCAGCGCCCACGCCAGCTTCACAC
P20	lolA F16A_R	GTGTGAAGCTGGCGTGGGCGCTGCTGACTTTATCC
P21	lolA R43L_F	GGCGATCTGTGGGTGAAACTTCCAAACTTATTCAACTG
P22	lolA R43L_R	CAGTTGAATAAGTTTGGAAGTTTCACCCACAGATCGCC
P23	lolA F47A_F	GATCTGTGGGTGAAACGTCCAAACTTAGCCAACTGGCATATGAC
P24	lolA F47A_R	GTCATATGCCAGTTGGCTAAGTTTGGACGTTTCACCCACAGATC
P25	lolA W49A_F	GAAACGTCCAAACTTATTCAACGCGCATATGACACAACCTGATG
P26	lolA W49A_R	CATCAGGTTGTGTCATATGCGCGTTGAATAAGTTTGGACGTTTC
P27	lolA F68A_F	GACGGTAAAACACTGTGGGCCTATAACCCGTTCGTTGAG
P28	lolA F68A_R	CTCAACGAACGGGTTATAGGCCCACAGTGTTTTACCGTC
P29	lolA F90A_F	CACCGGTAATACGCCGGCTATGCTGATTGCCCGC
P30	lolA F90A_R	GCGGGCAATCAGCATAGCCGGCGTATTACCGGTG
P31	lolA F90E_F	GCCACCGGTAATACGCCGGAGATGCTGATTGCCCGCAAC
P32	lolA F90E_R	GTTGCGGGCAATCAGCATCTCCGGCGTATTACCGGTGGC
P33	lolA F140A_F	GCACAATCCATCAGGCTAGCGCGGTGGAGCAG
P34	lolA F140A_R	CTGCTCCACCGCGCTAGCCTGATGGATTGTGC
P35	lolA F140E_F	GATGGCACAATCCATCAGGAGAGCGCGGTGGAGCAGGAC
P36	lolA F140E_R	GTCCTGCTCCACCGCGCTCTCCTGATGGATTGTGCCATC
P37	lolA S150A_F	GAGCAGGACGATCAGCGCGCCAGTTATCAACTGAAATC
P38	lolA S150A_R	GATTTCAGTTGATAACTGGCGCGCTGATCGTCCTGCTC
P39	lolA Y152A_F	GACGATCAGCGCAGCAGTGCTCAACTGAAATCCCAG
P40	lolA Y152A_R	CTGGGATTTCAGTTGAGCACTGCTGCGCTGATCGTC

P41	lolA Y152F_F	CGATCAGCGCAGCAGTTTTCAACTGAAATC
P42	lolA Y152F_R	GATTTCAGTTGAAAACTGCTGCGCTGATCG
P43	lolA S150A_Y152A_F	GGAGCAGGACGATCAGCGCGCCAGTGCTCAACTGAAATCCCAGC
P44	lolA S150A_Y152A_R	GCTGGGATTTCAGTTGAGCACTGGCGCGCTGATCGTCCTGCTCC

Table S5. List of plasmids.

Name	Description	Reference
pLDR8	*int* gene expression vector, helper plasmid	(8)
pLDR9	Cloning vector for integration into attB, kan resistant	(8)
pKD13	Kan cassette template for lambda red recombination	(9)
pSIM5	Expression of lambda red recombination genes	(10)
pET24-periLolC	Expresses LolC (residues 48-266) with a C-terminal His-tag	(11)
pET28-periLolC	Expresses LolC (residues 48-266) with a N-terminal, thrombin cleavable His-tag	(7)
pET28-LolA	Expresses LolA (residues 22-203) with a N-terminal, thrombin cleavable His-tag	(7)
pET28-LolA(R43L)	Expresses LolA R43L (residues 22-203) with an N-terminal, thrombin cleavable His-tag	This study
pET28-mLolB	Expresses mLolB (residues 23-207) with an N-terminal, thrombin cleavable His-tag	(7)
pCDFDuet	Expression vector	Novagen
pACYCDuet	Expression vector	Novagen
pCDF-LolA	Co-expresses LolA with a C-terminal Strep-tag (WSHPQFEK) separated by a GS linker	This study
pCDF-LolA-Pal_{WT}	Co-expresses LolA with a C-terminal Strep-tag (WSHPQFEK) separated by a GS linker and Pal with a C-terminal His-tag separated by a GS linker	This study
pCDF-LolA-$Pal_{WT(octa)}$	Co-expresses LolA with a C-terminal Strep-tag (WSHPQFEK) separated by a GS linker and Pal with a C-terminal TEV cleavage site (ENLYFQS) and an octahistidine-tag, each separated by a GS linker	This study
pCDF-LolA-$Pal_{TEV/FL}$	Co-expresses LolA with a C-terminal Strep-tag (WSHPQFEK) separated by a GS linker and Pal containing an internal TEV cleavage site (ENLYFQS) at position 13 in the mature sequence and a C-terminal hexahistidine-tag	This study
pCDF-LolA-$Pal_{TEV/FL(thrb)}$	Co-expresses LolA with a C-terminal Strep-tag (WSHPQFEK) separated by a GS linker and Pal containing an internal TEV cleavage site (ENLYFQS) at position 13 in the mature sequence and a C-terminal thrombin cleavable hexahistidine-tag	This study
pCDF-LolA(R43L)-$Pal_{TEV/FL2}$	Co-expresses LolA R43L with a C-terminal Strep-tag (WSHPQFEK) separated by a GS linker and Pal containing an internal TEV cleavage site (ENLYFQS) at position 28 in the mature sequence and a C-terminal hexahistidine-tag	This study
pAC80-LolA(XnY)	Expresses C-terminally strep-tagged LolA or indicated variant where residue X at position n mutated to residue Y	This study

Supplementary Movies

Movie S1. Representative electron density for the LolA-lipoprotein complex. The protein model is shown in green with its weighted $2|F_o|-|F_c|$ electron density map, represented as a gray mesh contoured at 1 σ. The lipoprotein ligand is shown in light orange with its difference omit map, represented as a blue mesh contoured at 3 σ. The polder omit map (12) of the lipoprotein ligand is shown in the last section of the movie, represented as a cyan mesh contoured at 3 σ.

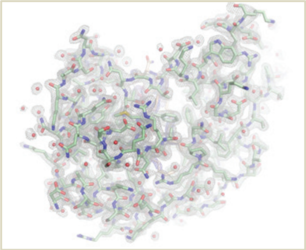

Movie S2. Molecular morph showing the sequential transition of LolA from apo to LolC-bound and lipoprotein-liganded conformations. *Left*, side-on view of LolA alternating between its conformation in isolation (1IWL), bound to LolC (burgundy, 6F3Z) and liganded to a lipoprotein (light orange, 7Z6W). For clarity, only the Hook of LolC is represented. *Right*, bottom-down view of the same morph in surface representation showing the cavity entrance of LolA.

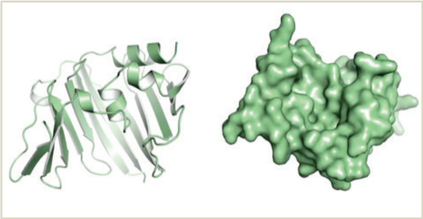

Movie S3. Representative electron density for the R43L LolA-lipoprotein complex. The protein model is shown in blue with its weighted $2|F_o|-|F_c|$ electron density map, represented as a gray mesh contoured at 1 σ. The lipoprotein ligand is shown in light orange with its difference omit map, represented as a blue mesh contoured at 3 σ. The polder omit map (12) of the lipoprotein ligand is shown in the last section of the movie, represented as a brown mesh contoured at 3 σ.

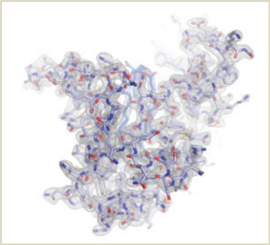

Movie S4. Residue-interaction network of R43L in the different LolA states. Hydrogen bonds involving R43L in LolA are shown as dashed lines while the protein transits from the apo (1IWL), LolC-bound (6F3Z) and lipoprotein-associated (7Z6W) state.

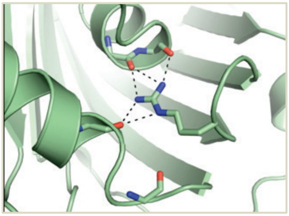

Supplementary Methods

Construction of strains and plasmids

Primer sequences and constructs used in this study are detailed in **Tables S4 and S5** respectively. To construct an arabinose-inducible *lolA* conditional knockout strain (BW49), the *lolA* locus including the ribosome binding site was amplified using primers P1 and P2 and cloned into the EcoRI-XbaI sites of pBAD18 (13). The region encompassing the *araC* gene, pBAD promoter, *lolA* and the downstream terminator was amplified using primers P3 and P4, digested XbaI-SacI and cloned into the integration vector pLDR9 digested with the same enzymes. The construct was integrated into the lambda *attB* site of *E. coli* BW25113 according to a previously described protocol (8). The native copy of *lolA* was replaced with a kanamycin resistance cassette by amplifying pKD13 with primers P5 and P6 using the λ Red recombinase system as described (9) except that pSIM5 (10) was used for recombinase expression. Deletions were confirmed by PCR of the gene locus with primers P7 and P8.

To enable complementation of BW49, plasmid pAC80-LolA was constructed. A synthetic fragment encoding the lac T5 promoter derived from pQE80 (Qiagen) followed by the RBS, C-terminally strep-tagged *lolA* gene and a transcriptional terminator was synthesized (IDT DNA). This was then amplified with primers (P9/P10) and introduced by Gibson assembly (14) into pACYCDuet digested with EcoNI and AvrII to remove the T7 promoter and multiple cloning sites. LolA variants were created by two-step PCR reactions. In the first step, two reactions consisting of P9/lolA mutant_R and P10/lolA mutant_F primers were set up with pAC80-LolA as a template. Following purification, a mixture of these products was used as a template for a final PCR using primers P9 and P10. The resultant product was then introduced by Gibson assembly into digested pACYCDuet as described for the wild-type gene.

For periplasmic co-expression of LolA and Pal, full-length LolA (residues 1-203) was amplified and an internal NdeI site simultaneously removed by a two-step PCR from *E. coli* M1655 genomic DNA using primers P11/P14 and P12/P13. The products were purified, mixed and used as a template for a subsequent PCR reaction using primers P11 and P12. Following digestion with BspHI-BamHI, this product was inserted into the first multiple cloning site (MCS) of pCDFDuet (Novagen) digested NcoI-BamHI, resulting in pCDF-LolA. The *pal* gene was amplified from MG1655 genomic DNA using primers P15/P16, digested NdeI-XhoI and inserted into the 2nd MCS of pCDF-LolA. The resultant vector pCDF-LolA-Pal$_{WT}$, encodes C-terminally Strep-tagged LolA and C-terminally His-tagged Pal. To enable the removal of the C-terminal His-tag on Pal, a fragment encoding a GS linker and TEV site (amino acid sequence: ENLYFQS) between the C-terminus of Pal and an octa-histidine tag was synthesized (IDT DNA) and cloned NdeI-XhoI into the 2nd MCS of pCDF-LolA resulting in pCDF-LolA-Pal$_{WT(octa)}$. To enable removal of the globular domain of Pal, constructs in which a TEV site was introduced at residues 34 and 49 of full-length Pal (residues 13 and 28, respectively, in the mature sequence) of full-length Pal were synthesized (IDT DNA) and cloned into the 2nd site of pCDF-LolA using NdeI-XhoI resulting in LolA-Pal$_{TEV/FL}$ and LolA-Pal$_{TEV/FL2}$ respectively. To permit removal of the His-tag from Pal$_{TEV/FL}$, a thrombin site was introduced immediately before the His-tag by Quikchange mutagenesis with

primers P17 and P18 resulting in pCDF-LolA-$Pal_{TEV/FL(thrb)}$. Plasmids pET28-LolA and pET28-mLolB expressing the mature domain of LolA (residues 22-203) and mLolB (residues 23-207) with an N-terminal His-tag were previously described (7). The R43L mutation was introduced into pCDF-LolA by Quikchange mutagenesis using primers P21 and P22. All constructs were verified by DNA sequencing (Source Bioscience).

Complementation assay of LolA variants

Strain BW49 bearing pAC80-LolA or the indicated variant was cultured in LB supplemented with 0.2% arabinose and appropriate antibiotics. The next day, 1 mL of overnight culture was centrifuged at 7000 *g* for 2 mins, washed twice in LB supplemented with 0.1% D-fucose and then diluted 1/10000 into fresh LB containing 0.1% fucose. Cells were then grown to an OD_{600} of 0.5, pelleted at 7000 *g*, washed twice in LB and then serial tenfold dilutions performed in LB. Dilutions were plated out on LB agar with no arabinose and grown overnight at 37 °C before imaging the next day. To assess expression level of the variant proteins, whole cell samples were resolved on SDS-PAGE, transferred to PVDF membrane, and immunoblotted with an anti-strep (IBA) and a dye conjugated Donkey anti-mouse secondary (Licor) antibodies. Immunoblots were revealed using an Odyssey Licor fluorescence imager.

Protein purification

Purification of wild-type LolA complexed with Pal

Cultures of *E. coli* C43 (DE3) carrying pCDF-LolA-$Pal_{WT(octa)}$, pCDF-LolA-$Pal_{TEV/FL}$ or pCDF-LolA-$Pal_{TEV/FL(thrb)}$ as appropriate were grown in 2YT media supplemented with 50 µg/mL streptomycin and 0.5% glycerol at 37 °C until an OD_{600} of 0.4 was attained. Protein expression was then induced with 1 mM IPTG and growth continued for a further 5 hours. Cells were pelleted at 3250 *g* for 20 min and carefully resuspended in TSE buffer (200 mM Tris pH 8.0, 1 mM EDTA, 20% sucrose) supplemented with EDTA-free mini protease inhibitor tablets (Roche). Lysozyme (0.5 mg/mL final concentration) was then added and cells incubated on ice for 1 hour. The resulting spheroplasts were removed by centrifugation at 20000 *g*, 4 °C, 30 minutes prior to clarification of the supernatant by centrifugation (1h at 115000 *g*, 4 °C). The resulting periplasmic fraction was then diluted with an equal volume of water to reduce the sucrose concentration to 10%, supplemented with 5 mM $MgCl_2$, 300 mM NaCl and 20 mM imidazole and loaded on a 5 mL FF Histrap column (GE Healthcare). The column was washed with 75 mL of 25 mM HEPES pH 7.5, 300 mM NaCl, 20 mM imidazole and then eluted with 10 mL of the same buffer containing 250 mM imidazole. Fractions were then dialyzed against 25 mM HEPES pH 7.5, 200 mM NaCl for 4 hours. To remove the His-tag, the protein was supplemented with 2 mM TCEP and cleaved with a 5:1 molar ratio of LolA-Pal complex:protease using TEV protease produced from plasmid pSH24-TEV (15). Thrombin cleavage used the Thrombin CleanCleave kit (Sigma) following the manufacturer's instructions. After digestion, the mixture was applied to 1 mL His trap FF column (GE Healthcare) with LolA-Pal complex found in the flow-through. Where required, a further purification step utilizing the C-terminal strep-tag on LolA was performed using Strep resin (Strep-Tactin XT 4Flow, IBA). Complexes were concentrated, snap frozen in liquid nitrogen and stored at -80 ºC.

Purification of R43L LolA-Pal complex
The complex was produced from the LolA R43L-Pal$_{TEV/FL}$ construct where the TEV cleavage site was introduced at position 49 of full-length Pal (position 28 in the mature sequence). Proteins were produced in a similar manner to the wild-type LolA-Pal complex but with the following modifications. *E. coli* cells were induced at an OD_{600} of 0.9 for 2h only and pelleted at 3250 *g* for 30 min. The spheroplast treatment was carried for 1h at RT, and proteins were desalted in 20 mM HEPES pH 7.5, 200 mM NaCl after IMAC. No TCEP was added to the protein and TEV cleavage was performed with a molar ratio of 1:35 protease: complex overnight at 4 ºC, before removing the His-tagged Pal mature domain and the TEV protease with an excess of Ni resin. The resultant complex containing LolA R43L associated with the 28 N-terminal residues of Pal was concentrated to 6.4 mg/mL, snap frozen in liquid nitrogen and stored at -80 ºC.

Transfer of lipoproteins from LolA to mLolB. LolA-Pal complexes from which the His-tag had been cleaved (see above) were mixed at a 2:1 ratio to 15 µM of His-tagged soluble mLolB (7) in a final volume of 200 µL in a buffer containing 25 mM HEPES pH 7.5, 200 mM NaCl. After 30 minutes at 37 °C, the mixtures were incubated with 100 µL of Ni-IMAC resin (Biorad) for 5 minutes in microbatch spin columns (Generon). The resin was washed four times with 400 µL of buffer prior to elution of bound proteins with 250 µL of buffer containing 250 mM imidazole. The elution fractions were analyzed on a 12% SDS-PAGE gels including purified mLolB and LolA-Pal complexes as references.

Preparation of spheroplasts and immunoblotting. *E. coli* C43 (DE3) cells carrying either empty pCDFDuet vector, pCDF-LolA-Pal$_{WT}$ or pCDF-LolA-Pal$_{TEV/FL}$ with a C-terminal His-tag on Pal were grown at 37 °C in LB supplemented with 100 µg/mL streptomycin. At OD_{600} 0.3-0.4, half of the cells were induced with 0.1 mM IPTG and grown for a further 75 minutes at 37 °C. Bacteria were pelleted by centrifugation at 3000 *g* for 20 min at 4 °C and then converted into spheroplasts by resuspending the cell pellets in 1.3 mL (final volume) of TSE buffer containing 0.5 M sucrose, 50 mM Tris pH 8.0 and 1 mM EDTA, supplemented with protease inhibitor cocktail (Roche) and 0.5 mg/mL lysozyme. After 30 min of incubation on ice, periplasmic extracts and outer membranes were separated from spheroplasts and intact cells by centrifugation at 16000 *g* for 30 min at 4 °C. Outer membranes were pelleted by centrifugation at 255000 *g* for 30 min at 4 °C from 1 mL supernatant and resuspended in 150 µL of 10 mM Tris pH 7.5 containing SDS-PAGE loading buffer. Spheroplast pellets were homogenized in 1 mL of 10 mM Tris pH 7.5 prior to a 15-fold dilution in buffer containing SDS-PAGE loading buffer (150 µL final volume).
Fractions were analyzed on SDS-PAGE, transferred to a PVDF membrane and probed with anti-His (Qiagen) and a dye-conjugated Goat anti-mouse secondary (LI-COR). LolB or inner membrane protein AcrA (16) were detected using polyclonal rabbit antibodies and dye conjugated Donkey anti-rabbit secondary antibody. Bands were visualized using a LI-COR Odyssey imaging system.

Crystallization and structure determination
All crystals were grown at 15 °C by the sitting-drop vapor-diffusion method over a reservoir of 80 µL in MRC 2-drop plates (Molecular Dimensions).

Wild-type LolA-lipoprotein complex

Complexes of LolA and lipoprotein, derived from the N-terminal 13 residues of mature Pal lipoprotein, were concentrated to 11 mg/mL and mixed at a 2:1 protein:reservoir ratio in 1 µL final volume. Initial hits were obtained in 2.1 M DL-Malic acid pH 7.0 but diffracting crystals were grown at pH 6.0. Crystals were cryoprotected in the reservoir solution supplemented with 20% glycerol before being flash frozen in liquid nitrogen. Diffraction data were collected on beamline I04-1 at Diamond synchrotron, indexed and reduced with iMosflm (17), scaled with Aimless (4). The structure was solved by molecular replacement using Phaser (18) and LolA (1UA8). The model was further improved by several rounds of Refmac (5) and manual building in Coot (19). The final structure was validated with Rampage (6) and Procheck (20). Density inside the cavity of LolA enabled the location of the lipoprotein three acyl chains and corresponding triacylated cysteine but not of the other residues. Structural data were collected for another eight crystal structures from this construct as well as a complex containing wild-type LolA and the N-terminal 28 residues of mature Pal lipoprotein; all revealed identical positioning of the lipoprotein acyl chains.

LolA R43L-lipoprotein complex

Complexes of LolA R43L and lipoprotein, derived from the N-terminal 28 residues of mature Pal lipoprotein (LolA R43L-Pal_{28}), were concentrated to 6.5 mg/mL and mixed at a 1:1 protein:reservoir ratio in 1 µL final volume. Diffracting crystals grew in 30% PEG3350, 50 mM bis-Tris pH 6.0 with seeds of crystals obtained in 10% 2-Propanol (v/v), 0.1 M HEPES pH 7.5 and 0.2 M NaCl. Crystals directly flash frozen in liquid nitrogen and data were collected under cryogenic conditions on beamline X06SA at SLS (Switzerland) on a EIGER 16M detector. The structure was solved as described for wild-type LolA-lipoprotein complex using the apo R43L LolA structure (2ZPD) for molecular replacement. As for the wild-type LolA complex, there was no density to model the protein sequence after the triacylated +1 cysteine.

Structure PDB depositions

Coordinates and structure factors were deposited in the Protein Data Bank under accession codes **7Z6W** (LolA wild-type – lipoprotein), and **7Z6X** (LolA R43L – lipoprotein).

Evaluation of A22 and MAC13243 inhibitors effect on Lol proteins

MAC13243 (Cambridge bioscience) and A22 (Sigma Aldrich) compounds were prepared in DMSO at 100 mM and tested at 0, 0.5, 1 or 2 mM in 2% DMSO final concentration.

Effect of inhibitors on LolA-Pal complex. *E. coli* phospholipid bilayer coated silica beads (100 µL), prepared as previously described (21), were incubated with 15 µM LolA-Pal complex from which the C-terminal His-tag of Pal had been removed and A22 or MAC13243 compound in a buffer containing 50 mM Tris pH 8.5, 200 mM NaCl and 2% DMSO supplemented with 50 µM bovine serum albumin (Thermofisher) to reduce non-specific binding in a final volume of 250 µL. After 30 min of incubation under gentle agitation, the mixture was centrifuged at 16000 *g* for 30 seconds, the supernatant removed and the beads washed three times with 1 mL of the same buffer before elution in 25 µL of 2% SDS, 0.5 mM EDTA, 1 mM TCEP, 6 M urea, 50 mM Tris pH 9.5, and bromophenol blue. For each fraction, 1 µL of the SDS preparation was loaded on a gradient SDS-PAGE gel before imaging with an Odyssey (LI-COR) system.

Effect of inhibitors on LolC-LolA association and LolA to LolB lipoprotein transfer. LolC-LolA interaction in the presence of A22 and MAC13243 was assessed as follows: tag-free LolA at 15 µM final concentration was pre-incubated with A22 or MAC13243 (0, 0.5, 1 or 2mM final concentrations) for 30 min in a buffer containing 25 mM HEPES pH 7.5, 150 mM NaCl and 2% DMSO. The same concentration of His-tagged LolC periplasmic domain (7) was added to the mixture and loaded in microbatch spin columns (Generon) with 100 µL nickel resin (Biorad) for 5 min. The resin was washed three times with 500 µL of the same buffer before elution of resin-bound proteins with 250 µL of buffer containing 250 mM of imidazole. Eluted proteins were analyzed on gradient SDS-PAGE gels, imaged with an Odyssey (LI-COR) system and the results normalized to the values obtained without compound. Transfer of Pal from LolA to LolB in the presence of A22 or MAC13243 inhibitor was assessed in a similar manner by pre-incubating His-tagged soluble LolB (7) with the compounds prior to addition of tag-free LolA-Pal complex.

Isothermal titration calorimetry (ITC)

ITC experiments were performed with a VP-ITC calorimeter (Malvern Panalytical) at 25 °C in 20 mM HEPES pH 7.5, 200 mM NaCl. Injections were 10 µL except the first which was 5 µL and occurred every 200 s until the syringe was empty. Cell stirring speed was 300 rpm, reference power 25 µcal/s and initial delay 60 s. Association of wild-type LolA or the R43L variant with mLolB was measured by injecting 250 to 500 µM of LolA from the syringe into the cell containing mLolB at 25 or 30 µM. Binding between the LolC periplasmic domain and LolA R43L was assessed at a protein concentration of 450 µM in the syringe and 40 or 45 µM in the cell. LolA wild-type at 300 µM was injected onto LolC periplasmic domain at 25 µM, while the reverse data are from (7). For each ITC run, a control experiment was performed by injecting protein into a cell containing only buffer. Corresponding data were then subtracted from the protein-protein interaction as a linear fit. Raw data were fitted with PEAQ-ITC (Malvern Panalytical) software using the single-site binding model for LolA wild-type or R43L interaction with mLolB and LolC while the two-site binding model was used for the LolA R43L-LolC data. Good fits were obtained for the biphasic data but inspection of reduced Chi-squared statistics by offsetting several values indicated that ΔH and $-T\Delta S$ parameters were poorly defined by the fit.

Fluorescence spectroscopy

Fluorescence measurements of the fluorescent fatty acid probe 11-(dansylamino)undecanoic acid (DAUDA, Cayman Chemical) was performed in a FluoroLog (Horiba) spectrometer at room temperature in a final volume of 1.2 mL using a 1 cm quartz cuvette. DAUDA stock solution was prepared at a concentration of 1 mM in DMSO. Fluorescence was recorded between 400 and 600 nm in 20 mM HEPES pH 7.5, 150 mM NaCl in the presence of 10 µM DAUDA, after excitation at 335 nm. His-tagged LolA WT or R43L and tag-free LolC periplasmic domain proteins were mixed in a LolC:LolA ratio of 2:1. Measurements were performed with LolA concentrations of 0, 5, 10, 15, 20, 30 and 40 µM after briefly stirring the solution with a magnet.

Molecular Docking

The protein-protein association between lipoprotein-bound LolA after removal of the ligand (7Z6W) and mLolB (1IWM, chain A) was predicted with the ClusPro 2.0 server (22). We selected the docking solution corresponding to the second most populated cluster according to ClusPro

clustering method, which was in good agreement with experimental data obtained by the Tokuda lab (2).

SI References

1. N. Buddelmeijer, The molecular mechanism of bacterial lipoprotein modification—How, when and why? *FEMS Microbiol. Rev.* **39**, 246–261 (2015).
2. S. Okuda, H. Tokuda, Model of mouth-to-mouth transfer of bacterial lipoproteins through inner membrane LolC, periplasmic LolA, and outer membrane LolB. *Proc. Natl. Acad. Sci. U. S. A.* **106**, 5877–82 (2009).
3. L. Pravda, *et al.*, MOLEonline: A web-based tool for analyzing channels, tunnels and pores (2018 update). *Nucleic Acids Res.* **46**, W368–W373 (2018).
4. P. R. Evans, G. N. Murshudov, How good are my data and what is the resolution? *Acta Crystallogr. Sect. D Biol. Crystallogr.* **69**, 1204–1214 (2013).
5. G. N. Murshudov, *et al.*, REFMAC5 for the refinement of macromolecular crystal structures. *Acta Crystallogr. Sect. D Biol. Crystallogr.* **67**, 355–367 (2011).
6. S. C. Lovell, *et al.*, Structure validation by C alpha geometry: phi,psi and C beta deviation. *Proteins-Structure Funct. Genet.* **50**, 437–450 (2003).
7. E. Kaplan, N. P. Greene, A. Crow, V. Koronakis, Insights into bacterial lipoprotein trafficking from a structure of LolA bound to the LolC periplasmic domain. *Proc. Natl. Acad. Sci. U. S. A.* **115**, E7389–E7397 (2018).
8. L. Diederich, L. J. Rasmussen, W. Messer, New cloning vectors for integration into the λ attachment site attB of the *Escherichia coli* chromosome. *Plasmid* **28**, 14–24 (1992).
9. K. A. Datsenko, B. L. Wanner, One-step inactivation of chromosomal genes in *Escherichia coli* K-12 using PCR products. *Proc. Natl. Acad. Sci. U. S. A.* **97**, 6640–6645 (2000).
10. S. Datta, N. Costantino, D. L. Court, A set of recombineering plasmids for gram-negative bacteria. *Gene* **379**, 109–115 (2006).
11. A. Crow, N. P. Greene, E. Kaplan, V. Koronakis, Structure and mechanotransmission mechanism of the MacB ABC transporter superfamily. *Proc. Natl. Acad. Sci.* **114**, 12572–12577 (2017).
12. D. Liebschner, *et al.*, Polder maps: Improving OMIT maps by excluding bulk solvent. *Acta Crystallogr. Sect. D Struct. Biol.* **73**, 148–157 (2017).
13. L. M. Guzman, D. Belin, M. J. Carson, J. Beckwith, Tight regulation, modulation, and high-level expression by vectors containing the arabinose P(BAD) promoter. *J. Bacteriol.* **177**, 4121–4130 (1995).
14. D. G. Gibson, *et al.*, Enzymatic assembly of DNA molecules up to several hundred kilobases. *Nat. Methods* **6**, 343–345 (2009).
15. S. Van Den Berg, P. Å. Löfdahl, T. Härd, H. Berglund, Improved solubility of TEV protease by directed evolution. *J. Biotechnol.* **121**, 291–298 (2006).
16. S. Lobedanz, *et al.*, A periplasmic coiled-coil interface underlying TolC recruitment and the assembly of bacterial drug efflux pumps. *Proc. Natl. Acad. Sci. U. S. A.* **104**, 4612–4617 (2007).
17. T. G. G. Battye, L. Kontogiannis, O. Johnson, H. R. Powell, A. G. W. Leslie, iMOSFLM: A new graphical interface for diffraction-image processing with MOSFLM. *Acta Crystallogr. Sect. D Biol. Crystallogr.* **67**, 271–281 (2011).
18. A. J. McCoy, *et al.*, Phaser crystallographic software. *J. Appl. Crystallogr.* **40**, 658–674 (2007).
19. P. Emsley, B. Lohkamp, W. G. Scott, K. Cowtan, Features and development of Coot. *Acta Crystallogr. Sect. D Biol. Crystallogr.* **66**, 486–501 (2010).
20. R. A. Laskowski, M. W. MacArthur, D. S. Moss, J. M. Thornton, PROCHECK: a program to check the stereochemical quality of protein structures. *J. Appl. Crystallogr.* **26**, 283–291 (1993).
21. P. J. Hume, D. Humphreys, V. Koronakis, *WAVE regulatory complex activation*, 1st Ed. (Elsevier Inc., 2014).
22. D. Kozakov, *et al.*, The ClusPro web server for protein-protein docking. *Nat. Protoc.* **12**, 255–278 (2017).
